# Identification of new 4-(6-oxopyridazin-1-yl)benzenesulfonamides as multi-target anti-inflammatory agents targeting carbonic anhydrase, COX-2 and 5-LOX enzymes: synthesis, biological evaluations and modelling insights

**DOI:** 10.1080/14756366.2023.2201407

**Published:** 2023-04-20

**Authors:** Waleed A. Badawi, Mahmoud Rashed, Alessio Nocentini, Alessandro Bonardi, Mohammad M. Abd-Alhaseeb, Sara T. Al-Rashood, Giri Babu Veerakanellore, Taghreed A. Majrashi, Eslam B. Elkaeed, Bahaa Elgendy, Paola Gratteri, Claudiu T. Supuran, Wagdy M. Eldehna, Mohamed Elagawany

**Affiliations:** a Department of Pharmaceutical Chemistry, Damanhour University, Damanhour, Buhaira, Egypt; b Pharmaceutical Medicinal Chemistry & Drug Design Department, Faculty of Pharmacy, Al-Azhar University, Cairo, Egypt; c Department of Neurofarba, Section of Pharmaceutical and Nutraceutical Sciences, University of Florence, Firenze, Sesto Fiorentino, Italy; d Department of Neurofarba, Section of Pharmaceutical and Nutraceutical Sciences, Laboratory of Molecular Modeling Cheminformatics & QSAR, University of Florence, Polo Scientifico, Firenze, Sesto Fiorentino, Italy; e Department of Pharmacology and Toxicology, Damanhour University, Damanhour, Buhaira, Egypt; f Department of Pharmaceutical Chemistry, College of Pharmacy, King Saud University, Riyadh, Saudi Arabia; g Center for Clinical Pharmacology, Washington University School of Medicine and University of Health Sciences and Pharmacy, St. Louis, MO, USA; h Department of Pharmacognosy, College of Pharmacy, King Khalid University, Abha, Saudi Arabia; i Department of Pharmaceutical Sciences, College of Pharmacy, AlMaarefa University, Riyadh, Saudi Arabia; j Chemistry Department, Faculty of Science, Benha University, Benha, Egypt; k Department of Pharmaceutical Chemistry, Kafrelsheikh University, Kafrelsheikh, Egypt

**Keywords:** Analgesic, Synthesis, Pyridazinone, Sulphonates, Molecular modelling

## Abstract

Multiple inhibitions of CA, COX-2 and 5-LOX enzymes has been recognised as a useful strategy for the development of anti-inflammatory drugs that can avoid the disadvantages of using NSAIDs alone. Here, we report new pyridazine-based sulphonamides (**5a-c** and **7a-f**) as potential multi-target anti-inflammatory candidates. First, the furanone heterocycle in the dual CA/COX-2 inhibitor Polmacoxib was replaced with the pyridazinone one. Then, a hydrophobic tail was appended through benzylation of the 3-hydroxyl group of the pyridazinone scaffold to afford benzyloxy pyridazines **5a-c**. Furthermore, the structures were adorned with the polar sulphonate functionality, in pyridazine sulphonates **7a-f**, that are expected to be engaged in interactions with the hydrophilic half of the CA binding sites. All of the disclosed pyridazinones were tested for inhibitory activities against 4 *h*CA isoforms **(I**, **II**, **IX**, and **XII**), as well as against COX-1/2, and 5-LOX. Furthermore, *in vivo* anti-inflammatory and analgesic effects of pyridazinones **7a** and **7b** were examined.

## Introduction

Carbonic anhydrases (EC 4.2.1.1) are metalloenzymes that have a metal ion in their active site.^1^ CAs were thoroughly studied for decades and were classified as a superfamily of enzymes with eight gene families or classes to date.^2^ There are basically several cytosolic forms (CA I-III, CA VII), four membrane-bound isozymes (CA IV, CA IX, CA XII, and CA XIV), a mitochondrial form (CA V), and a secreted CA isozyme, CA VI.^3^ Carbonic anhydrase is involved in diverse physiological and pathological processes including lipogenesis, gluconeogenesis, and tumorigenicity.^4^ One of the major biochemical reactions involved in proton generation in resting tissues is mediated by carbonic anhydrase (CA) enzymes.^5^ They are known for their ability to catalyse the reversible hydration of carbon dioxide (CO_2_ + H_2_O ⟺ H^+^ + HCO_3_^−^) and have also been extensively demonstrated in a plethora of physiological events at the cellular and tissue levels.^6^ Numerous CA isoforms have evolved because uncatalyzed transformation is unable to meet the physiological cells' needs.^7^^,^^8^

At the cellular level, overexpression of CA increases the concentration of ions (H^+^ and HCO_3_), and since bicarbonate ions are necessary cellular components that are immediately recovered, local extracellular acidosis is established straight away. A decrease in tissue pH is associated with inflammatory pain, and carbonic anhydrases (CAs) are primarily responsible for proton production in tissues.^9^^,^^10^ It has been well-established that the intensity of inflammation and pain-associated symptoms in diverse inflammatory disorders, such as rheumatoid arthritis, is inversely correlated with tissue pH values.^9^^,^^10^ Due to their role in the regulation of pH by reversibly catalysing the conversion of CO_2_ to bicarbonate and protons, CAs have been recently reported to be implicated in diverse inflammation reactions.^11–13^ In particular, Cimaz et al.^14^ reported that hCA IX and XII isoforms, which are widely expressed in hypoxic malignancies, are also over-expressed in the inflamed synovium of individuals with juvenile arthritis.

Lipid mediators, which include the classic eicosanoids, prostaglandins (PGs), and leukotrienes (LTs), are potent inflammatory mediators produced by local cell-type specific arachidonic acid metabolism. However, the arachidonic acid metabolism is carried out by the cyclooxygenase (COX) and lipoxygenase (LOX) families of enzymes, leading to the synthesis of prostaglandins and leukotrienes. Pain, inflammation, asthma, and allergies are all treated with drugs that target these enzymes.^15^ Dual COX-2 and 5-LOX enzyme inhibition has recently been recognised as a useful strategy for the development of anti-inflammatory drugs that can get around the disadvantage of using NSAIDs alone.^16–19^

**Figure 1.**
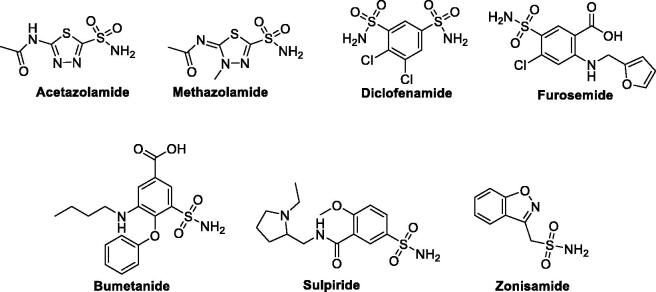
Examples of clinically used sulphonamide-based drugs.

Over the last few decades, sulphonamide-based small molecules were highlighted as an important class that is utilised for the management of many illnesses. Currently, there are more than 20 drugs in clinical usage, such as Acetazolamide (AAZ), Methazolamide, Diclofenamide, Furosemide, Bumetanide, Sulpiride, and Zonisamide (Figure 1). It has been well-established that the sulphonamide functionality and its isosteric groups (sulfamate and sulfamide) is a crucial element for the CA and selective COX-2 inhibitory actions.^20^

**Figure 2.**
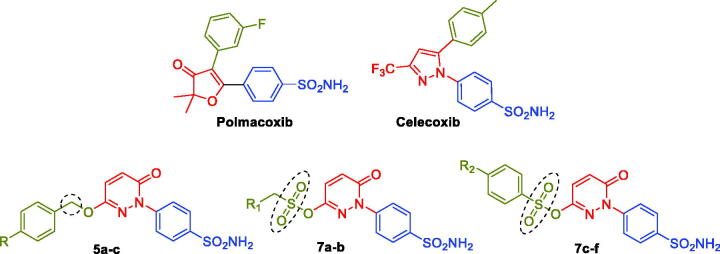
Chemical structures of the dual CA/COX-2 inhibitors **Polmacoxib** and **Celecoxib**, as well as the target pyridazinones **5a-c**, and **7a-f**.

**Polmacoxib** (Acelex, Figure 2) is a sulphonamide-tethered first-in-class dual CA/COX-2 inhibitor that was approved in 2015 by the Korean Ministry of Food and Drug Safety (MFDS) for the management of osteoarthritis.^21–24^ Interestingly, the CA inhibitory activity has been reported for several sulphonamide-tethered COX-2 inhibitors such as Celecoxib (Figure 2).^23^^,^^25^^,^^26^ Besides, a proof-of-concept report has revealed that the combination of the fragments of nonsteroidal anti-inflammatory drugs (NSAIDs) and CA inhibitors could improve the effectiveness of drugs used to treat ache symptoms linked to inflammatory disorders including rheumatoid arthritis (RA).^27^

As a privileged scaffold, the pyridazinone motif has attracted a lot of interest in the realm of drug discovery. Due to its diverse range of biological actions and potential therapeutic uses, the pyridazinone moiety has been the focus of extensive investigations.^28^ Of particular interest, diverse pyridazinone-based molecules have been reported for their anti-inflammatory^29–35^ and carbonic anhydrase inhibitory^36^^,^^37^ activities.

In this report, we developed new pyridazinone-based sulphonamide derivatives (**5a-c** and **7a-f**, Figure 2) as potential multi-target anti-inflammatory candidates, using the multi-target strategy.^38–40^ The *h*CA, COX-2, and 5-LOX enzymes, which have been implicated in the inflammatory process, are anticipated to be efficiently inhibited by the designed pyridazinone-based sulphonamides (**5a-c** and **7a-f**). Furthermore, the *in vivo* anti-inflammatory effects for the target pyridazinones were examined.

The design of the target pyridazinone sulphonamides is based on the bioisosteric replacement of the furanone heterocycle in the dual CA/COX-2 inhibitor Polmacoxib with the pyridazinone one, while keeping the benzenesulfonamide motif directly attached to the heterocycle. The incorporated sulfamoyl functionality is anticipated to anchor with the catalytic Zn^2+^ in the CA binding site, and also promotes COX-2 selectivity *via* hydrogen bonding within the hydrophilic sub-pocket in the COX-2 binding site.

In addition to the bioisosteric approach followed in the design of the new ligands, a hydrophobic tail was appended to the structure in an attempt to boost the inhibitory activity and selectivity towards *h*CA isoforms IX and XII. This was achieved through benzylation of the 3-hydroxyl group of the pyridazinone scaffold to afford the first set of the target benzyloxy pyridazine compounds **5a-c** (Figure 2). In addition, the structures were adorned with the polar sulphonate functionality that is expected to be engaged in interactions within the hydrophilic half of the CAs binding site. This was achieved through sulfonation of the 3-hydroxyl group with either alkyl or aromatic sulphonyl chlorides to produce the corresponding alkyl and aromatic pyridazine sulphonates **7a-b** and **7c-f**, respectively.

## Results and discussion

### Chemistry

**Scheme 1.**
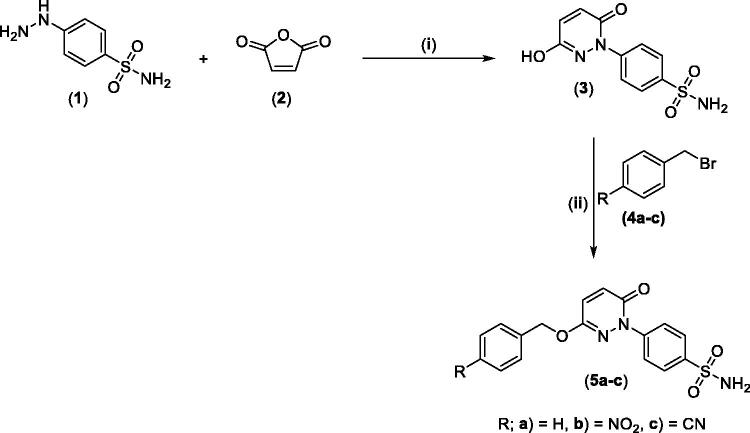
General Synthesis of pyridazine derivatives (**2** and **5a-c**); Reagents and conditions: (**i**) H_2_O, reflux, overnight; (**ii**) Potassium carbonate, DMF, stirring at 5 °C, 3 h.

**Scheme 2.**
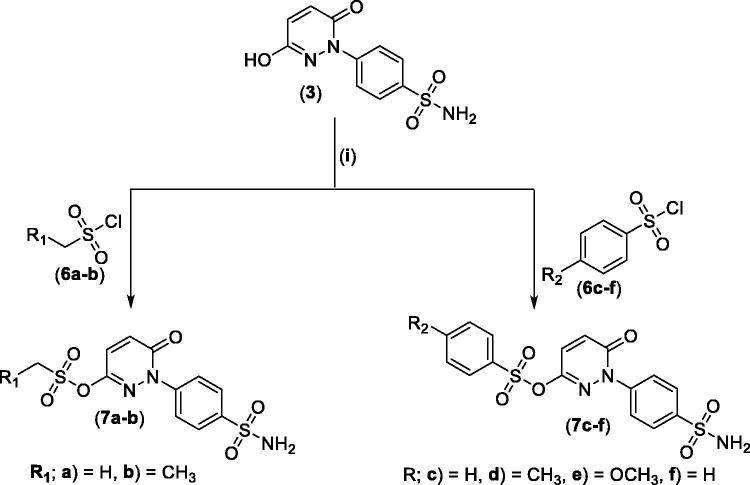
General Synthesis of pyridazine derivatives (7**a-f**); Reagents and conditions: (**i**) Pyridine, stirring at 5 °C, 1 h.

The synthesis of the target pyridazine derivatives is depicted in Schemes 1 and 2. A total of 10 compounds (**3**, **5a-c**, **and 7a-e**) were synthesised, all of them novel molecules, not previously described in the literature. The syntheses followed known procedures previously described somewhere else.^41–43^ The target compounds were obtained in an excellent isolated yield.

Reacting commercially available 4-hydrazineylbenzenesulfonamide (**1**) with maleic anhydride (**2**) under reflux conditions in water resulted in intermediate **3** in an excellent yield (92%). Next, we turned our attention to the reactivity of 4–(3-hydroxy-6-oxopyridazin-1(6*H*)-yl)benzenesulfonamide (**3**) towards an alkylation reaction, which is a very powerful tool for introducing chemical diversity. Compounds **5a-c** were accessible by an optimised alkylation procedure by reacting various benzyl bromide derivatives (**4a-c**) with intermediate (**3**) to afford the corresponding pyridazine derivatives in excellent yields (Scheme 1).

For the second set of the target pyridazine derivatives (7**a-f**), the synthesis was performed by reacting intermediate **3** with different aliphatic (**6a-b**) and aromatic (**6c-f**) sulphonyl chlorides in pyridine as base and solvent. The corresponding sulphonate derivatives (**7a-b** and **7c-f**) were formed in excellent yields after stirring at 5 °C for after 1h (Scheme 2).

### Biological evaluation

#### Carbonic anhydrase inhibition

Using the stopped-flow carbon dioxide hydration assay^44^ all the newly synthesised pyridazines (**3**, **5a-c** and **7a-f**) reported here and the standard CA inhibitor acetazolamide (AAZ) were evaluated for their inhibitory effects against hCA isoforms (I, II, IX, and XII). The tested CA isoforms were suppressed to varying degrees by the pyridazine-based benzenesulfonamides (PBS) described here, and the inhibition data are presented in Table 1. The structure-activity relationship (SAR) was obtained based on the inhibition results as *K*_I_ values for the synthesised analogues.

**Table 1.** *In vitro* inhibition data of hCA I, II, IX and XII with pyridazine derivatives (**3**, **5a-c** and **7a-f**) by the stopped flow kinetic assay using AAZ as a reference drug.

Compd.	R	*K*_I_ (nM)^a^			
		CA I	CA II	CA IX	CA XII
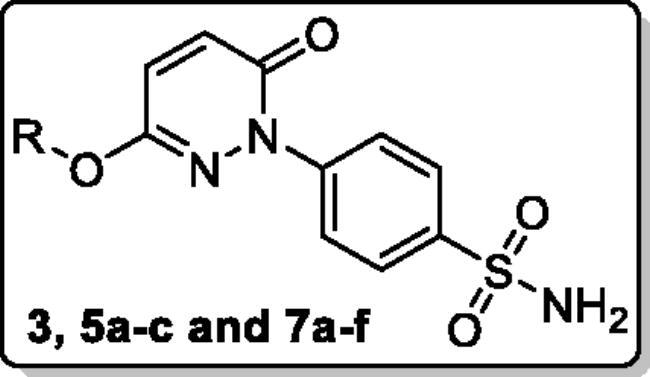					
**3**	H	23.5	55.8	45.1	5.3
**5a**	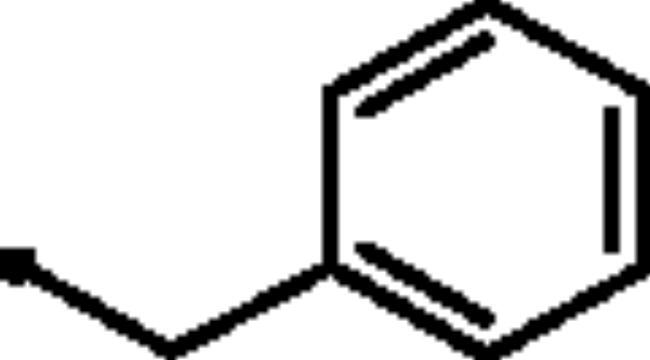	98.3	5.3	14.8	32.2
**5b**	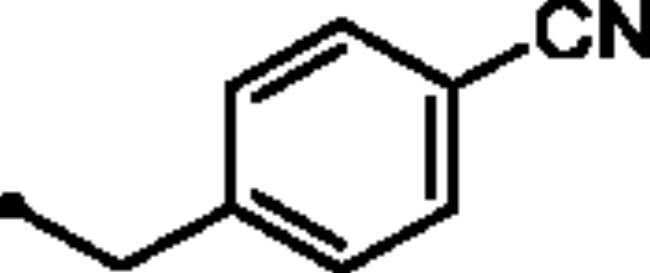	116.3	37.1	12.3	26.1
**5c**	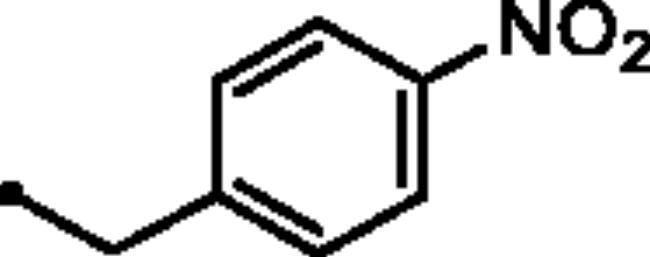	221.5	106.4	4.9	18.4
**7a**	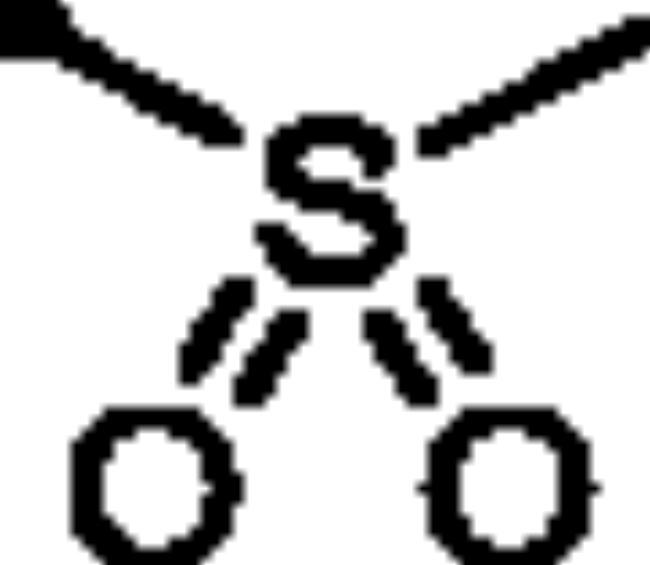	48.3	42.2	52.3	13.3
**7b**	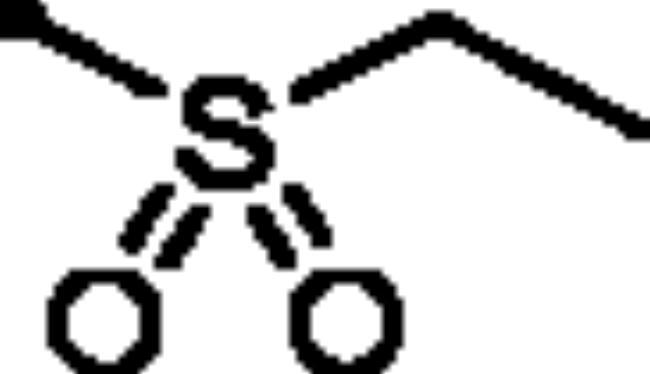	52.6	79.1	58.1	17.2
**7c**	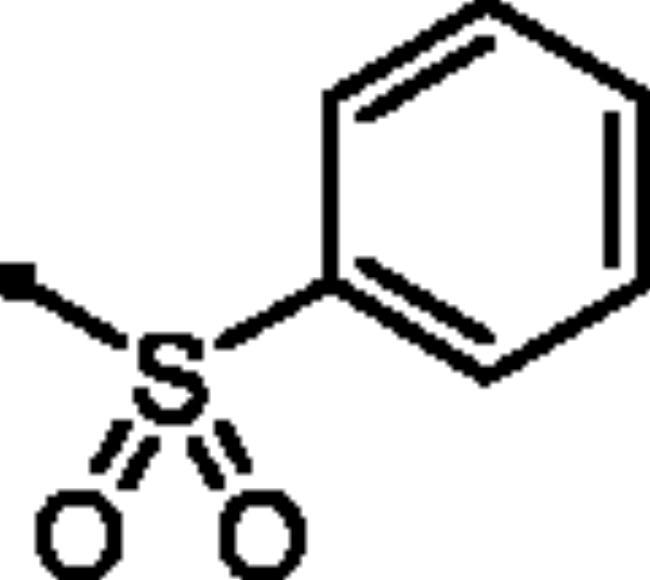	185.9	34.2	19.4	49.7
**7d**	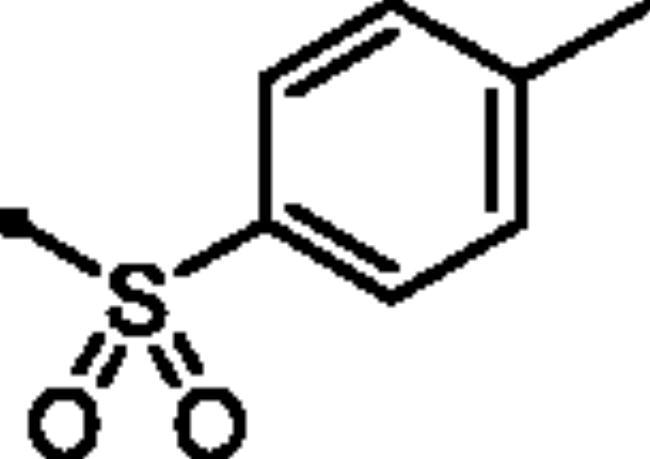	123.5	19.2	22.8	42.6
**7e**	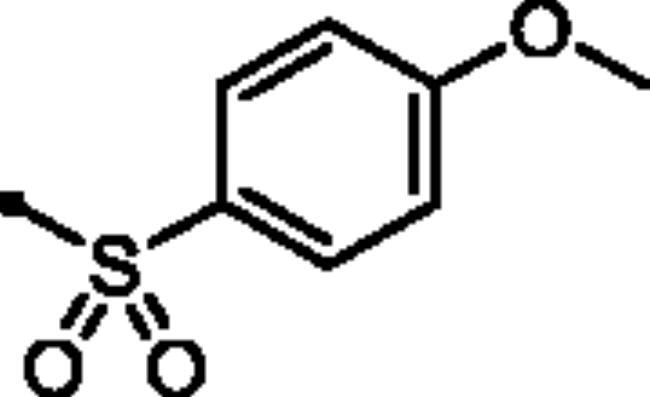	362.8	88.2	30.1	35.9
**7f**	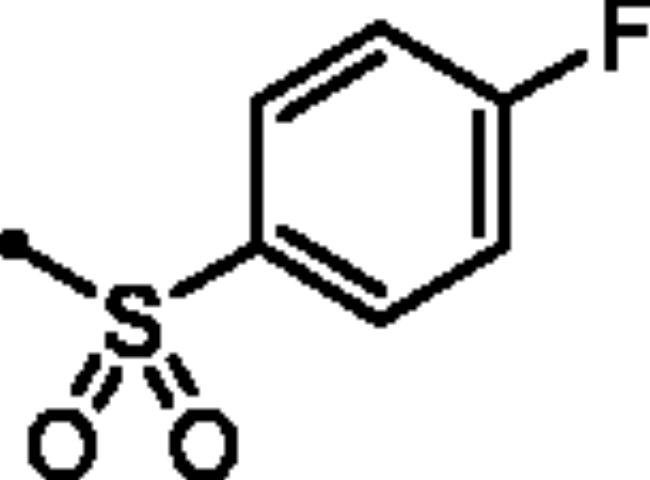	165.8	31.6	6.4	8.7
**AAZ**	–	250	12.5	25.0	5.7

^a^Means from three different assays, by a stopped flow technique.

The cytosolic hCA I isoform was suppressed by all pyridazine-tethered sulphonamide compounds (**3**, **5a-c** and **7a-f**) herein reported with inhibition constants (*K*_I_s) ranging from low to moderate nanomolar concentrations (i.e. between 23.5 nM and 362.8 nM). The *in vitro* kinetic data disclosed that the unsubstituted pyridazine derivative **3** is the most potent inhibitor within the tested compounds against the hCA I isoform (*K*_I_ = 23.5 nM), followed by pyridazines **7a** and **7b** (*K*_I_s = 48.3 and 52.6 nM, respectively). Moderate inhibition potencies, with *K*_I_ values spanning between 98.3–185.9 nM were obtained for pyridazines **5a**, **5b**, **7d**, and **7c**. Compound **7e** was the least potent inhibitor (*K*_I_= 362.8 nM) compared to the standard CAI AAZ (*K*_I_= 250 nM). In general, both benzylation (series **5**; *K*_I_: 98.3 − 221.5 nM) and sulfonation (series **7**; *K*_I_: 48.3 − 362.8 nM) of the key intermediate 3-hydroxy-6-oxopyridazin **3** (*K*_I_ = 23.5 nM) did not improve the inhibitory activities against hCA I. For the benzyloxy pyridazine series **5a-c**, the substitution of the pendant phenyl motif led to a decrease in activity towards CA I (**5b** and **5c**: *K*_I_ = 116.3 and 221.5 nM *vs.* **5a**: *K*_I_= 89.3 nM). For the sulphonate series **7**, the alkyl sulphonates **7a** and **7b** (*K*_I_ = 48.3 and 52.6 nM, respectively) possessed better inhibitory activity than the aryl sulphonates **7c-f** (*K*_I_ = 185.9, 123.5, 362.8 and 165.8 nM, respectively).

Regarding the inhibitory activity towards hCA II isoform, all the examined pyridazine-based sulphonamide derivatives (**3**, **5a-c** and **7a-f**) exerted good activity with inhibition constants in the range of 5.3 − 106.4 nM (Table 1). Only the benzyloxy pyridazine derivative **5a** was inhibited hCA II isoform at the single-digit nanomolar range (*K*_I_ = 5.3 nM). Moreover, the benzyloxy derivative **5a** as well as the sulphonate derivatives **7c**, **7d** and **7f** showed moderate activities towards hCA II isoform with inhibition constants equal 37.1, 34.2, 19.2 and 31.6 nM, respectively. Generally, benzylation (series **5**) and sulfonation (series **7**) of the key intermediate 3-hydroxy-6-oxopyridazin **3** (*K*_I_ = 55.8 nM) have enhanced the hCA II inhibitory effect, except for pyridazines **5c**, **7b** and **7e** (*K*_I_ = 106.4, 79.1 and 88.2 nM, respectively) (Table 1).

The synthesised pyridazine-based sulphonamide derivatives (**3**, **5a-c** and **7a-f**) all successfully suppressed the cancer-related hCA IX isoform (*K*_I_: 4.9 − 58.1 nM) as indicated in Table 1. The benzyloxy derivative **5c** and the sulphonate derivative **7f** were superior to the other derivatives and showed single-digit inhibitory activities (*K*_I_ = 4.9 and 6.4 nM, respectively) against hCA IX isoform. In addition, pyridazine derivatives **5a**, **5b**, **7c** and **7d** showed potent activity with *K*_I_ values equal to 14.8, 12.3, 19.4 and 22.8 nM, respectively. It is important to emphasise that the benzyloxy-tethered derivatives **5a-c** (*K*_I_: 4.9 − 14.8 nM) and the aryl sulphonate derivatives **7c-f** (*K*_I_: 6.4 − 58.1 nM) displayed much enhanced inhibitory activity than the intermediate 3-hydroxy-6-oxopyridazin **3** (*K*_I_ = 45.1 nM) against hCA IX, whereas the alkyl sulphonate derivatives **7a-b** (*K*_I_ = 52.3 and 58.1 nM, respectively) showed less activity than compound **3**.

In terms of the inhibitory effects against the second cancer-related hCA XII isoform tested here, all compounds (**3**, **5a-c** and **7a-f**) showed activities in the low nano-molar range (5.3 − 49.7 nM). Among the synthesised pyridazines, derivatives **3** and **7f** showed superior single-digit inhibition constants equal to 5.3 and 8.7 nM, respectively. Besides, pyridazine sulphonamides **5c**, **7a** and **7b** exerted effective hCA XII inhibitory effect (*K*_I_s = 18.4, 13.3 and 17.2 nM respectively). Notably, pyridazine **3** (*K*_I_ = 5.3 nM) demonstrated greater potency than its benzyloxy (series **5**; *K*_I_: 18.4 − 32.2 nM) and sulphonates (series **7**; *K*_I_: 8.7 − 49.7 nM) analogs. Also, it is worth mentioning that the substitution of the terminal phenyl moiety in both series **5** and **7** resulted in an enhancement for the hCA XII inhibitory action; compounds **5b-c** (*K*_I_s = 26.1 (**7b**) and 18.4 (**7c**) nM) vs. compound **5a** (*K*_I_ = 32.2 nM), and compounds **7d-f** (*K*_I_s = 42.6 (7d), 35.9 (**7e**) and 8.7 (**7f**) nM) vs. compound **7a** (*K*_I_ = 49.7 nM) (Table 1).

#### *In vitro* COX-1/COX-2 and LOX inhibitory activities

In this study, further evaluation of the anti-COX-2 effect of the pyridazine-based sulphonamide derivatives (**3**, **5a-c** and **7a-f**) was carried out, in addition to evaluation of the anti-COX-1 activity in order to assess the COX-2 selectivity of the synthesised pyridazines. Celecoxib (selective COX-2 inhibitor) and Indomethacin (non-selective inhibitor) were utilised as reference COX inhibitors. IC_50_ values for the inhibition of COX-1 and COX-2, as well as the selectivity index (IC_50_ of COX-1/IC_50_ of COX-2) are listed in Table 2. The COX-2 enzyme was effectively inhibited by the newly reported pyridazine-based sulphonamide derivatives (**3**, **5a-c**, and **7a-f**), with IC_50_ values in the sub-micromolar range ranging from 0.05 to 0.14 µM, whereas COX-1 was weakly inhibited with IC_50_ values spanning between 5 and 12.6 µM.

**Table 2.** IC_50_ values for the *in vitro* COX-1/2 and LOX inhibition, as well as COX SI values of the pyridazine-based sulphonamide derivatives **3**, **5a-c** and **7a-f**.

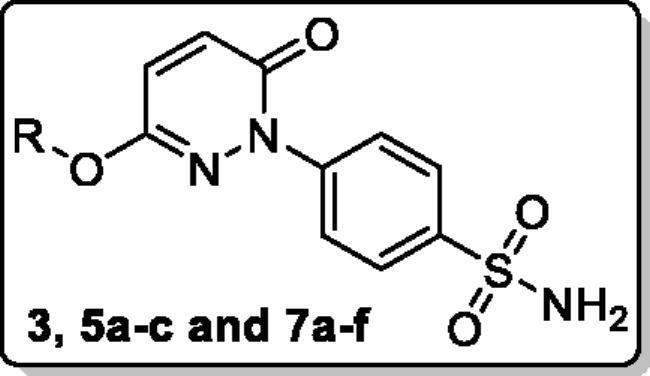				
Compd.	R	IC_50_ µM^a^		SI COX-1/ COX-2^b^
		COX-1	COX-2	
**3**	H	11.5	0.07	167.7
**5a**	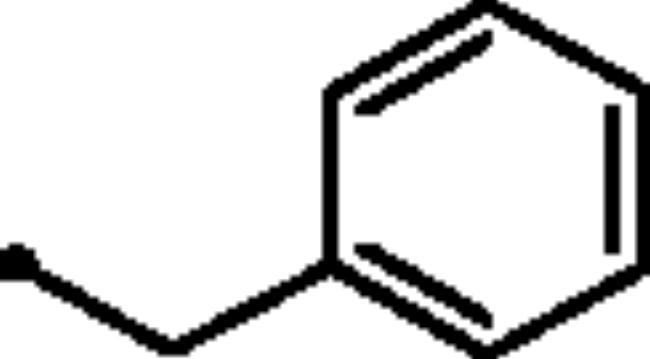	5	0.11	46
**5b**	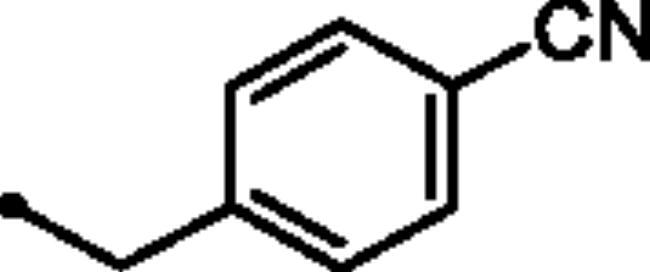	9.5	0.14	67.9
**5c**	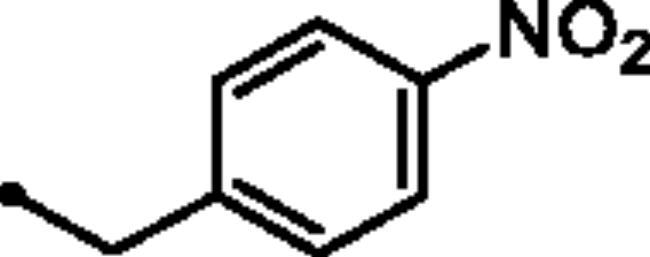	6.5	0.07	92.9
**7a**	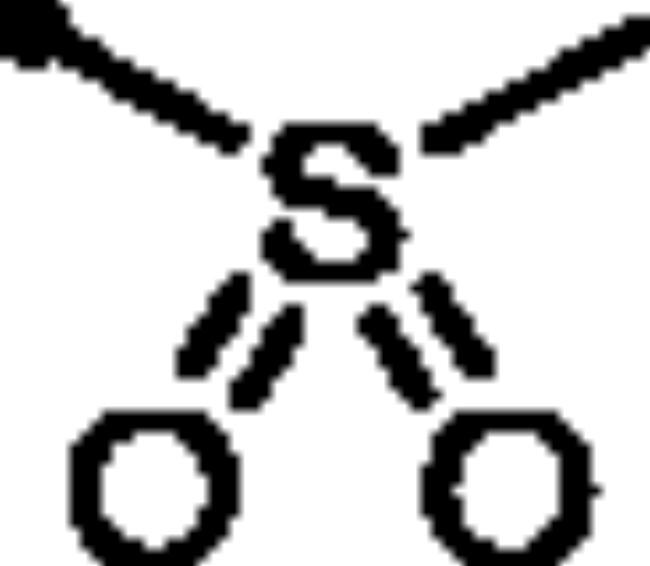	10.4	0.05	208
**7b**	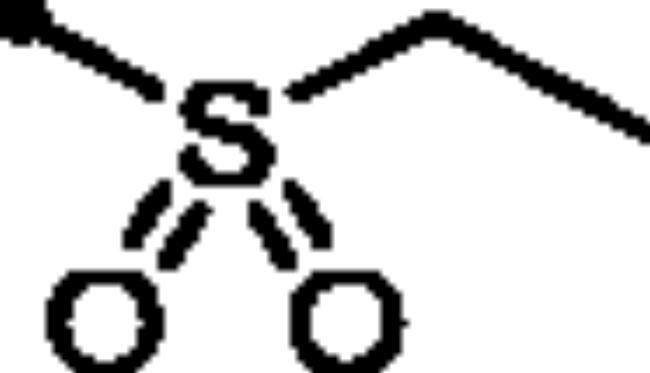	12.6	0.06	210
**7c**	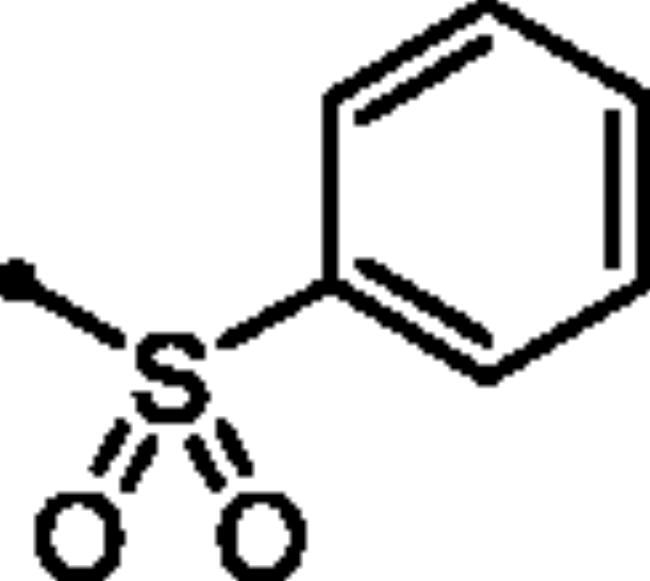	8.4	0.08	106
**7d**	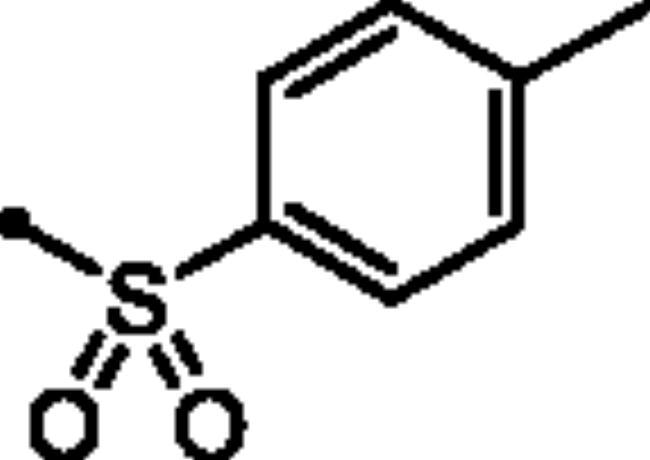	8.4	0.08	105.9
**7e**	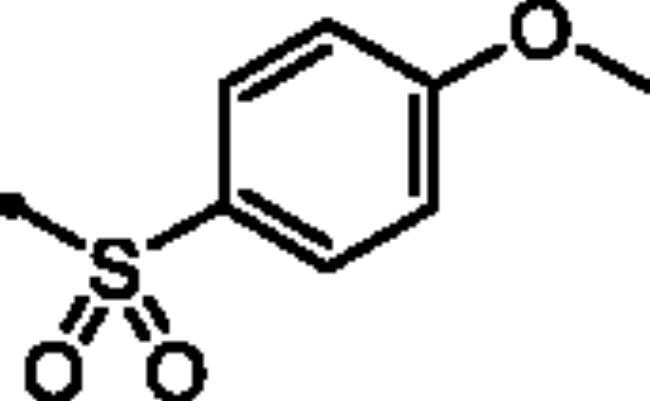	6.5	0.09	73.1
**7f**	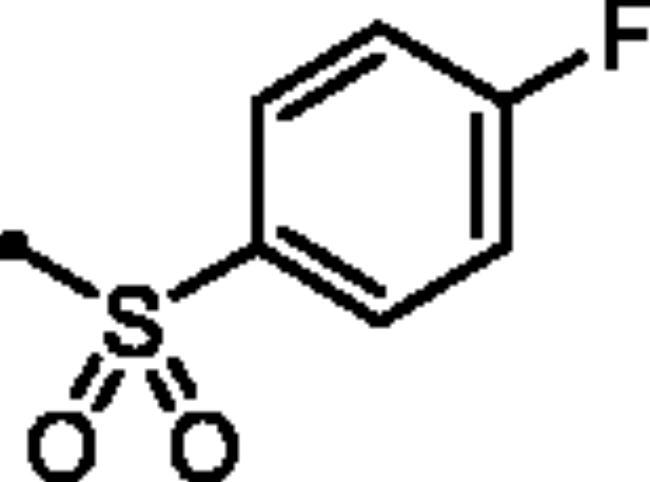	8.5	0.11	81
**Celecoxib**	–	14.5	0.05	313
**Indomethacin**	–	0.099	0.08	1.3

^a^IC_50_ values are expressed as the mean of three replicates with a standard deviation less than 10% of the mean. ^b^Selectivity index (SI)= IC_50_ (COX-1)/IC_50_ (COX-2).

The methanesulfonate and ethanesulfonate pyridazine derivatives (**7a** and **7b**) exerted the best COX-2 inhibitory effect (IC_50_ = 0.05 and 0.06 µM, respectively) among the tested derivatives. Both **7a** and **7b** were equipotent to the selective COX-2 inhibitor Celecoxib (IC_50_ = 0.05 µM) (Table 2). Moreover, pyridazines **3**, **5c**, **7c**, **7d** and **7e** showed potent activity towards COX-2 (IC_50_ = 0.07, 0.07, 0.8, 0.08 and 0.09 µM for **3**, **5c**, **7c**, **7d** and **7e**, respectively).

Concerning the selectivity of the target pyridazines towards COX-2 enzyme over COX-1, the findings highlighted that the pyridazine sulphonates (**7a** and **7b**) demonstrated the highest COX-2 selectivity index of 208 and 210, respectively (Table 2). In addition, the remaining pyridazine derivatives showed considerable selectivity towards COX-2 enzyme.

Additionally, in this study, the *in vitro* inhibitory activity of the pyridazine-based sulphonamide derivatives **3**, **5a-c**, and **7a-f** against the LOX enzyme was evaluated (Table 3).

**Table 3.** Inhibition data of LOX enzyme with pyridazine-based sulphonamide derivatives **3**, **5a-c** and **7a-f**.

Comp.	**IC_50_** (μM)^a^
**3**	2
**5a**	7
**5b**	5
**5c**	7
**7a**	3
**7b**	2.5
**7c**	4
**7d**	3.5
**7e**	5
**7f**	6.5
**Zileuton**	3.5

^a^Mean from 3 different assays.

The tested pyridazines displayed potent to moderate activity against LOX enzyme with IC_50_ range of 2 – 7 μM. The most potent LOX inhibitors in this work were found to be 3-hydroxy-6-oxopyridazine **3** (IC_50_ = 2 μM) as well as methanesulfonate **7a** (IC_50_ = 3 μM) and ethanesulfonate pyridazine derivatives **7b** (IC_50_ = 2.5 μM) that exerted better activity than the reference LOX inhibitor Zileuton (IC_50_ = 3.5 µM). It is worth noting that the best COX-2 inhibitors in this study were pyridazine sulphonates (**7a** and **7b**) (Table 2). As a result, both compounds **7a** and **7b** appear to be promising dual COX-2 and 5-LOX inhibitors.

#### In vivo biological evaluations

##### Analgesic activities

The acetic acid writhing test in mice has been used in this study to examine the analgesic properties of the most potent and selective COX-2 inhibitors (pyridazine sulphonates **7a** and **7b**).^45^ As shown in Table 4, the frequency of writhing was counted as a pain marker.

**Table 4.** Analgesic impact of the tested methanesulfonate and ethanesulfonate pyridazines **7a** and **7b** by the use of acetic acid writhing test in mice.

Comp.	Number of writhing (20 min) (Mean ± SD)	Analgesic activity (% protection)
7a	13 ± 0.5*	67.5%
7b	14 ± 1.3*	65.0%
Diclofenac	12 ± 1.9*	70.0%
Celecoxib	16 ± 0.9*	60.0%
Control	40 ± 2.9	---

The value is expressed as mean ± SD (*n* = 5). *Significantly different from the control group.

A significant reduction in the total number of writhing was observed as a direct result of treatment with both pyridazine sulphonates **7a** and **7b** (Number of writhing = 13 and 14, respectively) in comparison to the control group (Number of writhing = 40), which highlights the analgesic effect of **7a** and **7b**. It’s interesting to note that pyridazines **7a** and **7b** had stronger analgesic effects than celecoxib (Table 4).

##### Anti‐inflammatory activity

Carrageen-induced rat paw edoema protocol was exploited to evaluate the anti-inflammatory effects of the pyridazine sulphonates **7a** and **7b** as reported by Winter *et al*.^46^ using celecoxib and diclofenac as reference anti-inflammatory drugs. Table 5 displays the measured percentage change of paw height.

**Table 5.** Results of the carrageen-induced paw edoema assay that was used to investigate the effects of the pyridazine sulphonates **7a** and **7b** on paw height.

Comp.	FIF% change in paw height (Mean ± SD)				Anti-inflammatory activity (% inhibition of edoema)
	0 h	1 h	2 h	3 h	3 h
7a	0.52 ± 0.04	0.46 ± 0.09	0.44 ± 0.05	0.34 ± 0.05*	56%
7b	0.5 ± 0.07	0.44 ± 0.09	0.58 ± 0.04	0.48 ± 0.03*	38.5%
Diclofenac	0.52 ± 0.04	0.28 ± 0.04	0.32 ± 0.04	0.32 ± 0.04*	59%
Celecoxib	0.5 ± 0.07	0.44 ± 0.09	0.52 ± 0.04	0.48 ± 0.04*	38.5%
Control	0.56 ± 0.05	0.68 ± 0.04	0.64 ± 0.05	0.78 ± 0.08*	---

The value is expressed as mean ± SD (*n* = 5). *Significantly different from the control group.

The tested molecules **7a** and **7b** exerted significant (*p* < 0.05) anti-inflammatory activities *via* reducing the paw height and thus reduction of the paw edoema at 3 h in comparison to the control group (Table 5). The % inhibition of edoema of **7a** and **7b** were 56, and 38.5, respectively. Both compounds were superior to celecoxib with rapid onset of action after 1 h, and sustained duration until the third hour after the administration of the compound. Based on these findings, pyridazine **7a** could be a good anti-inflammatory candidate.

##### Inflammatory mediator’s measurements

Based on the encouraging anti-inflammatory and analgesic properties of pyridazine sulphonates **7a** and **7b** stated above, an ELISA assay was used to further assess the serum level of two inflammatory mediators namely, tumour necrosis factor-alpha (TNF-α) and interleukin one beta (IL-1β).

**Figure 3.**
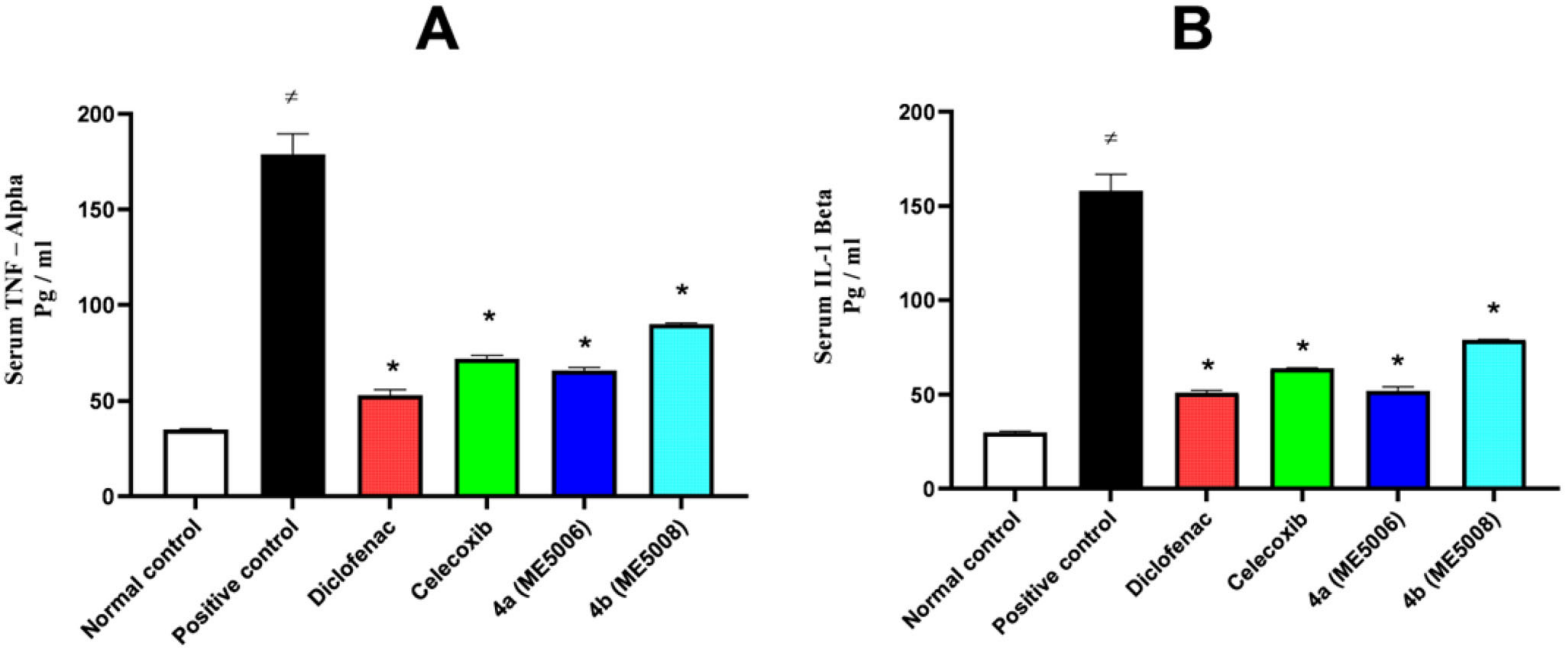
Serum level of the inflammatory mediators (TNF-α and IL-1β) after paw edoema test. The value is expressed as mean ± SD (*n* = 5). *Significantly different from the positive control group. A) Tumour necrosis factor – alpha (TNF-α). B) Interleukin one beta (IL-1β).

As can be seen in Figure 3, the levels of the inflammatory mediators in the positive control group were significantly higher at the end of the paw edoema test as compared to the levels in the normal control group. On the other hand, the studied sulphonates **7a** and **7b** significantly lowered TNF-α and IL-1β levels when compared to the positive control group.

##### Ulcerogenic effects

The degree of inflammation or ulceration of the examined pyridazine sulphonates (**7a** and **7b**) in the gastric tissues was evaluated and confirmed by histopathological examinations, following the previously described protocol.^47^

**Figure 4.**
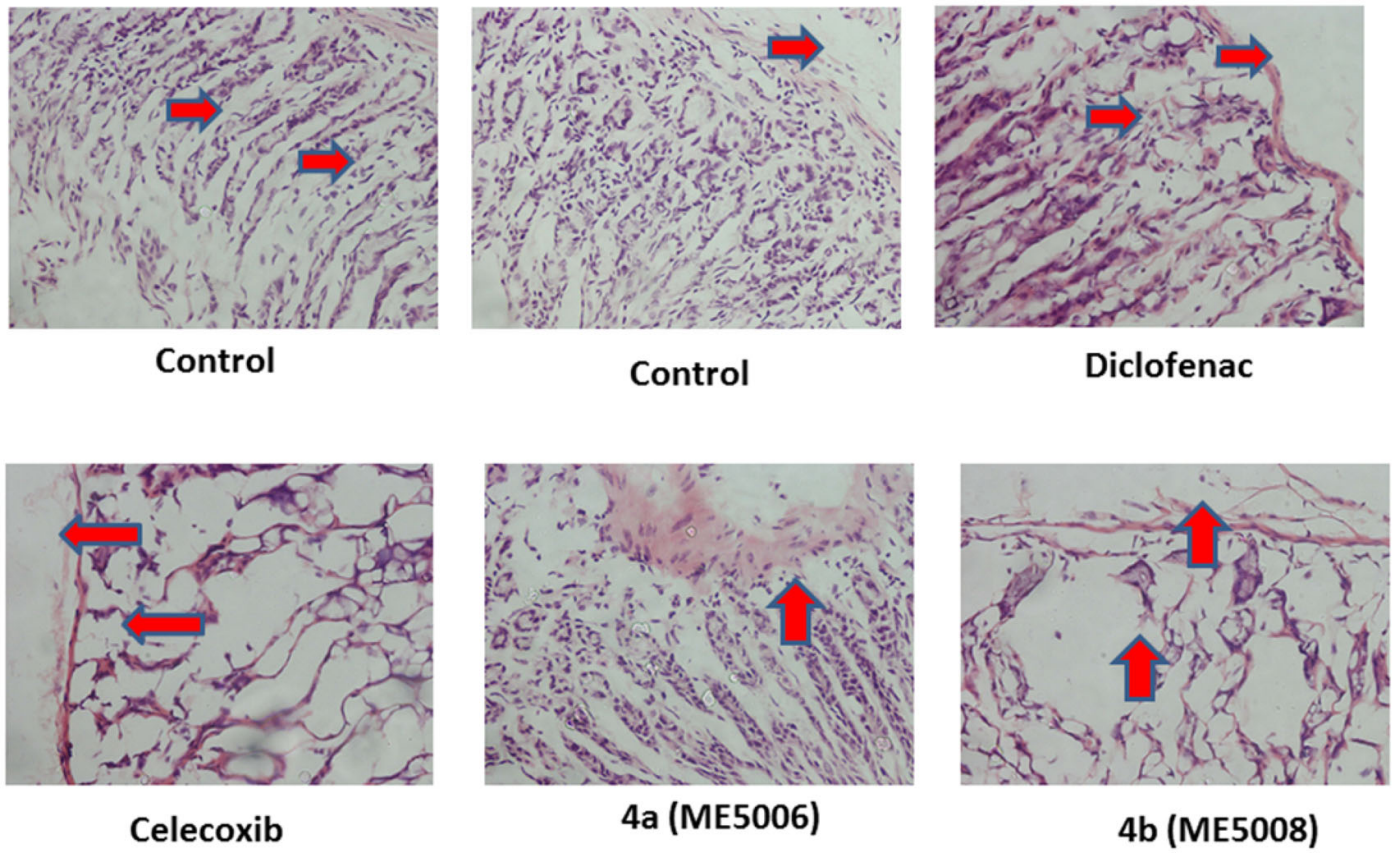
Ulcerogenic effects of the tested pyridazine sulphonates (**7a** and **7b**), diclofenac and celecoxib on the gastric tissue of rats. Histopathological examination was performed using H & E stain and the magnification power of the images was 40X. The red arrows indicated the normal or damaged parts.

As shown in Figure 4, the examination of the fasted rat stomach disclosed normal texture for pyridazine **7a** as well as the reference drug celecoxib. On the other hand, pyridazine **7b** displayed a mild inflammation as presented in Figure 4 (cf. red arrows). Diclofenac, also, showed inflammation and damage to the layers of the stomach (red arrows), which indicated some ulcerogenic side effects. These data suggested the safety profile of pyridazine **7a** on the stomach tissues.

### Molecular modelling

The most selective pyridazines against the tumour-associated isoforms CA IX and XII *versus* the ubiquitous cytosolic enzymes CA I and II, **5c** and **7f**, were investigated *in silico* to predict their binding mode within the active site of these four isozymes.

**Figure 5.**
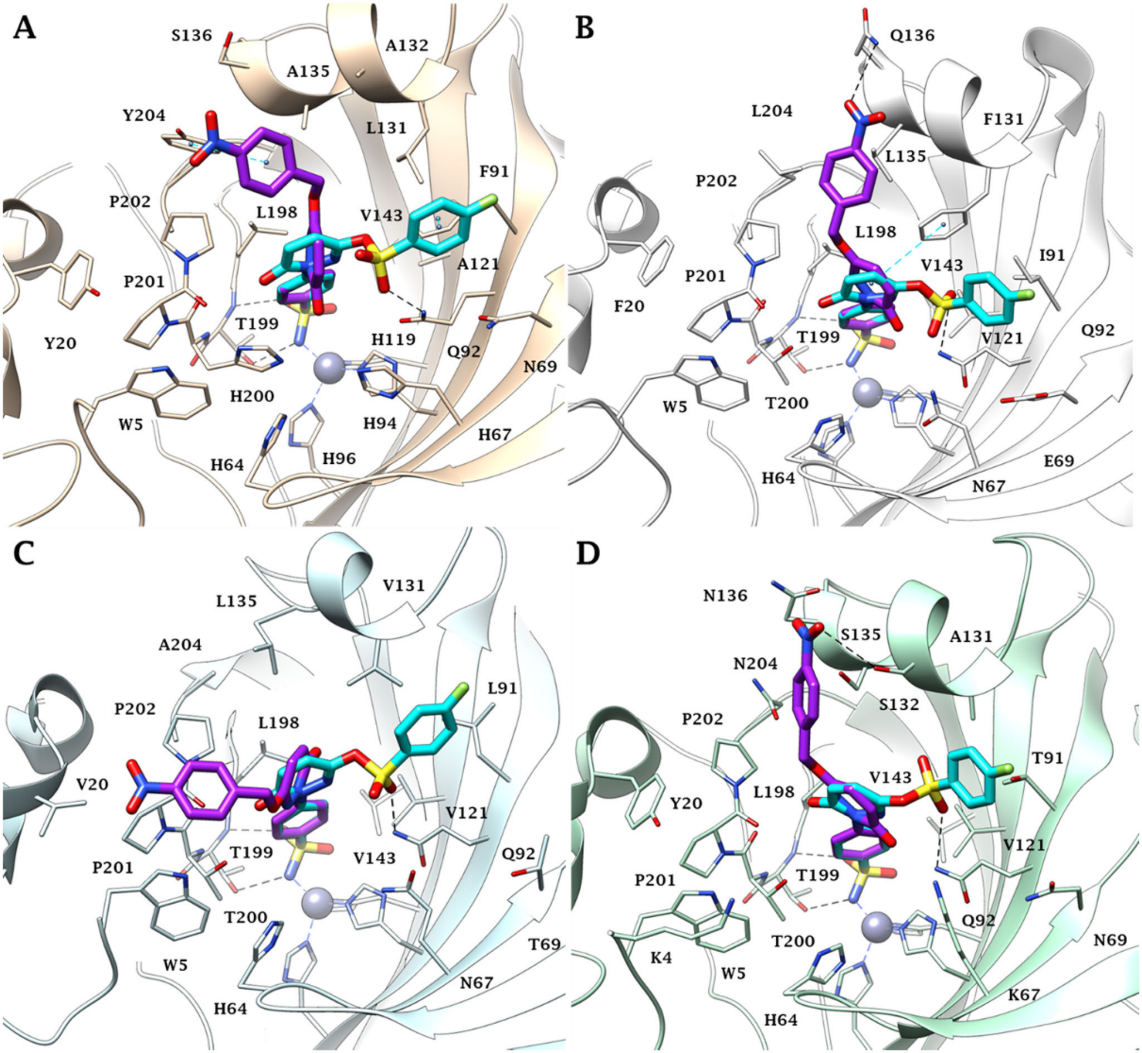
Predicted binding mode of **5c** (purple) and **7f** (cyan) within A) CA I, B) CA II, C) CA IX and D) CA XII active site.

As expected, all docking poses showed that the benzenesulfonamide moiety bonded to the zinc ion with the deprotonated nitrogen atom of the sulphonamide group (SO_2_NH^−^) in a tetracoordinated geometry. Moreover, the sulphonamide-metal coordination is stabilised by two H-bonds with the side chain OH and the backbone NH of T199, and by hydrophobic interactions that occur between the benzene ring and hydrophobic residues such as V143, L198 and W205 (Figure 5). In all four enzyme isoforms (CA I, II, IX, XII), the 4-nitrophenyl moiety was oriented towards a lipophilic cleft where the mutation of some amino acid residues occurs among the different CA isoforms, is responsible for the different sizes of the pocket. Consequently, the positioning and stabilisation of the ligands are different. In the CA I, the lipophilic area is lined by Y20, L131, A132, A135, P201, P202 and Y204. Here the 4-nitrophenyl moiety of **5c** engages in vdW contacts with A132, P202 and Y204, and a π-π stacking interaction with the Y204 side chain (Figure 5(A)). In CA II the Y204L, L131F and S136Q mutations allow the NO_2_ group to orient towards the Q136 and to establish an H-bond with the side chain NH_2_ (Figure 5(B)). The ligand is further stabilised by the hydrophobic contacts with F131, G132, L135 and L204 and by π-π stacking contact involving the aromatic pyridazin-3(2*H*)-one linker and the F131 side chain.

As a result of the CA I (or II)/IX mutations at Y20V (CA I/IX), Y204A (CA II/IX) and F131V in CA IX, the aryl pendant extends towards V20 making vdW interactions with this residue and W5, P201 and P202 (Figure 5(C)). In CA XII, the nitro group faces the same area as in CA II. However, due to the mutation Q136N (CA II/XII) and the shorter length of the amino acidic side chain, the group forms an H-bond with the side chain OH of S132 (Figure 5(D)). The larger lipophilic pocket of the tumour-associated isoforms (CA IX and XII) compared to the off-target (CA I and II) ones, allows **5c** a good fit and complementarity to the binding site, which is probably the reason for its better inhibitory profile observed experimentally.

In contrast to **5c**, the tail of **7f** is oriented towards the hydrophilic half of all investigated active sites. In particular, the sulphonic linker engages in an H-bond with the side chain NH_2_ of the conserved residues Q92 in CA I, II, IX and XII (Figure 5). The steric hindrance of F91 in CA I and, to less extent, F131 in CA II, led the 4-fluorophenyl group to move much closer to the hydrophilic residues N69 and E69 of the enzyme isoforms (Figures 5(A and B)). Moreover, the aromatic ring form π-π stacking interactions with F91 (CA I), while the pyridazin-3(2*H*)-one linker of **7f** forms π-π stacking interaction with the F131 side chain in CA II. Similarly, the larger lipophilic area of the tumour-associated isoforms allows the aryl moiety to engage many contact points with the targets CA IX/CA XII (L91/T91, V121, V131/A131 and Q92), thus binding a larger surface as compared to the other enzyme isoforms that might account for the activity profile (i.e. CA IX = CA XII > CA II ≫ CA I).

## Conclusion

We present the design and synthesis of a novel set of pyridazinone-based sulphonamide derivatives (**5a-c** and **7a-f**) as multi-carbonic anhydrase, COX-2, and 5-LOX inhibitors with the primary objective of developing new effective multitarget anti-inflammatory candidates. The furanone heterocycle in the dual CA/COX-2 inhibitor Polmacoxib was replaced with the pyridazinone one while keeping the benzenesulfonamide motif directly attached to the heterocycle. Then, a hydrophobic tail was appended through benzylation of the 3-hydroxyl group of the pyridazinone scaffold to afford benzyloxy pyridazine compounds **5a-c**. Also, the structures were adorned with the polar sulphonate functionality, in pyridazine sulphonates **7a-f**, that expected to be engaged in interactions with the hydrophilic half of all the CA active site. Most the synthesised pyridazine-based sulphonamides successfully suppressed the examined carbonic anhydrase isoforms in a variable degree; *K*_I_ ranges: 23.5 − 362.8 nM, 5.3 − 106.4 nM, 4.9 − 58.1 nM and 5.3 − 49.7 nM for CA I, II, IX and XII respectively, also, they effectively inhibited the COX-2 enzyme with IC_50_ values in the sub-micromolar range ranging from 0.05 to 0.14 µM, whereas COX-1 was weakly inhibited with IC_50_ values spanning between 5 and 12.6 µM. In particular, the methanesulfonate and ethanesulfonate pyridazine derivatives (**7a** and **7b**) exerted the most potent (IC_50_ = 0.05 and 0.06 µM) and selective (SI = 208 and 210) COX-2 inhibitory activity. Moreover, the tested pyridazines displayed potent to moderate activity against LOX enzyme with IC_50_ range of 2–7 μM. Further *in vivo* investigations for pyridazine sulphonates **7a** and **7b** revealed their ability to reduce the total number of writhing in mice, the rat paw edoema, and the serum levels of the inflammatory mediators (TNF-α and IL-1β), which highlights their analgesic and anti-inflammatory activities.

## Experimental

### Chemistry

#### General remarks

^1^H and ^13^C NMR spectra were collected by a Bruker Avance 400 MHz NMR spectrometer at 400 MHz (^1^H), or 101 MHz (^13^C), respectively. Chemical shifts are reported in parts per million (ppm) relative to the deuterated solvent, i.e. DMSO, δ ^1^H: 2.49 ppm; ^13^C: 39.7 ppm. PLC-HRMS analyses were performed on reverse phase gradient using Agilent (Santa Clara, CA) analyses series binary pump (G1312B), waters XTerra MS C18 (3.5 um; 2.1 × 150 mm) + Phenomenex C_18_ security guard column (2 × 4 mm) using 0.2% acetic acid in H_2_O/methanol as mobile phases; wavelength = 254 nm; and mass spectrometry was done with 6220 Agilent (Santa Clara, CA) TOF in electrospray ionisation (ESI) mode with the positive and negative method in both Profile and Centroid mode.

#### 4–(3-Hydroxy-6-oxopyridazin-1(6H)-yl)benzenesulfonamide (3)

In 50 ml screw cap vial, maleic anhydride **2** (2 g, 20.4 mmol) was added to a solution of 4-hydrazineylbenzenesulfonamide **1** (4.56 g, 20.4 mmol) in boiling water (20 ml). The reaction mixture was refluxed overnight, and then cooled to room temperature. The produced solid was collected by filtration, washed with hot water and dried to yield the desired compound **3** as a pale-yellow powder (92%); ^1^H NMR (400 MHz, DMSO-*d_6_*) *δ* 11.47 (s, 1H, OH), 7.96 − 7.86 (m, 2H), 7.86 − 7.77 (m, 2H), 7.44 (s, 2H, SO_2_NH_2_), 7.20 (d, *J* = 9.8 Hz, 1H), 7.05 (d, *J* = 9.8 Hz, 1H); ^13^C NMR (100 MHz, DMSO-*d_6_*) *δ* 157.86 (C = O), 153.16, 143.83, 142.49, 134.13, 128.19, 126.05, 125.54. HRMS *m/z* for C_10_H_9_N_3_O_4_S [M + Na]^+^. Calcd 290.020598, found 290.020620.

#### General procedure for the synthesis of benzyloxy pyridazine compounds (5a-c)

The appropriate benzyl bromide derivative **4a-c** (0.432 mmol) was added drop-wise to a solution of 4–(3-hydroxy-6-oxopyridazin-1(6*H*)-yl)benzenesulfonamide **3** (0.393 mmol) and potassium carbonate (0.432 mmol) in DMF (1 ml) at 5 °C, the reaction mixture was allowed to stir for 3 h and monitored with TLC. The reaction mixture was poured into cold water and extracted with ethyl acetate (3 × 15 ml). The organic layer was dried over anhydrous Na_2_SO_4_, filtered, and evaporated under reduced pressure to yield pyridazines **3a-c**.

##### 4–(3-(Benzyloxy)-6-oxopyridazin-1(6*H*)-yl)benzenesulfonamide (5a)

White powder (89%); ^1^H NMR (400 MHz, DMSO-*d_6_*) *δ* 7.89 (q, *J* = 8.8 Hz, 4H), 7.51 − 7.33 (m, 8H), 7.13 (d, *J* = 9.8 Hz, 1H), 5.24 (s, 2H); ^13^C NMR (100 MHz, DMSO-*d_6_*) *δ* 157.97 (C=O), 152.40, 143.77, 142.52, 136.02, 134.18, 128.45, 128.24, 127.87, 126.09, 125.15, 68.59. HRMS *m/z* for C_17_H_15_N_3_O_4_S [M + Na]^+^ calcd 380.057548, found 380.067428.

##### 4–(3-((4-Nitrobenzyl)oxy)-6-oxopyridazin-1(6*H*)-yl)benzenesulfonamide (5b)

White powder (91%); ^1^H NMR (400 MHz, DMSO-*d_6_*) *δ* 8.34 − 8.21 (m, 2H), 7.95 − 7.68 (m, 6H), 7.50 − 7.38 (m, 3H), 7.16 (d, *J* = 9.8 Hz, 1H), 5.41 (s, 2H); ^13^C NMR (100 MHz, DMSO-*d_6_*) *δ* 157.97, 152.07, 147.15, 143.94, 143.65, 142.57, 134.33, 128.63, 127.73, 126.10, 125.13, 123.59, 67.28. HRMS *m/z* for C_17_H_14_N_4_O_6_S [M + Na]^+^ calcd 425.052626, found 425.052865.

##### 4–(3-((4-Cyanobenzyl)oxy)-6-oxopyridazin-1(6*H*)-yl)benzenesulfonamide (5c)

White powder (90%);^1^H NMR (400 MHz, DMSO-*d_6_*) *δ* 7.90 (dd, *J* = 8.5, 6.9 Hz, 4H), 7.86 − 7.81 (m, 2H), 7.70 − 7.64 (m, 2H), 7.43 (d, *J* = 10.1 Hz, 3H), 7.15 (d, *J* = 9.8 Hz, 1H), 5.35 (s, 2H); ^13^C NMR (100 MHz, DMSO-*d_6_*) *δ* 157.98, 152.13, 143.67, 142.57, 141.85, 134.29, 132.42, 128.47, 127.79, 126.11, 125.14, 118.73, 110.74, 67.56. HRMS *m/z* for C_18_H_14_N_4_O_4_S [M + Na]^+^ calcd 405.062797, found 405.062819.

#### General procedure for the synthesis of sulphonate pyridazine compounds (7a-f)

The appropriate sulphonyl chloride derivative **6a-f** (0.374 mmol) was added to a solution of 4–(3-hydroxy-6-oxopyridazin-1(6*H*)-yl)benzenesulfonamide **3** (0.374 mmol) in pyridine (1 ml) at 5 °C and stirred for 1 h. The mixture was poured into 2 N HCl solution and then extracted with ethyl acetate (3 × 15 ml). The combined organic layers were washed with water and brine. The organic layer was dried over anhydrous Na_2_SO_4_, filtered and evaporated under reduced pressure to yield compounds **7a-f**.

##### 6-Oxo-1–(4-sulfamoylphenyl)-1,6-dihydropyridazin-3-yl methanesulfonate (7a)

White powder (94%); ^1^H NMR (400 MHz, DMSO-*d_6_*) *δ* 7.95 (d, *J* = 8.6 Hz, 2H), 7.83 (d, *J* = 8.6 Hz, 2H), 7.65 (d, *J* = 9.9 Hz, 1H), 7.49 (s, 2H), 7.32 (d, *J* = 9.9 Hz, 1H), 3.61 (s, 3H); ^13^C NMR (100 MHz, DMSO-*d_6_*) *δ* 158.38, 146.14, 143.53, 142.88, 134.91, 129.62, 126.32, 125.88, 39.21. HRMS *m/z* for C_11_H_11_N_3_O_6_S_2_ [M + Na]^+^ calcd. 367.998148, found 367.998341.

##### 6-Oxo-1–(4-sulfamoylphenyl)-1,6-dihydropyridazin-3-yl ethanesulfonate (7b)

White powder (95%); ^1^H NMR (400 MHz, DMSO-*d_6_*) *δ* 7.95 (d, *J* = 8.5 Hz, 2H), 7.85 − 7.78 (m, 2H), 7.69 − 7.60 (m, 1H), 7.49 (s, 2H), 7.31 (dd, *J* = 9.9, 0.8 Hz, 1H), 3.75 (q, *J* = 7.3 Hz, 2H), 1.39 (t, *J* = 7.3 Hz, 3H); ^13^C NMR (100 MHz, DMSO-*d_6_*) *δ* 158.35, 146.10, 143.51, 142.88, 134.87, 129.71, 126.34, 125.82, 46.30, 7.97. HRMS *m/z* for C_12_H_13_N_3_O_6_S_2_ [M + Na]^+^ calcd. 382.013798, found 382.013848.

##### 6-Oxo-1–(4-sulfamoylphenyl)-1,6-dihydropyridazin-3-yl benzenesulfonate (7c)

White powder (92%); ^1^H NMR (400 MHz, DMSO-*d_6_*) *δ* 8.08 − 7.99 (m, 2H), 7.88 (dd, *J* = 8.6, 2.2 Hz, 3H), 7.84 − 7.79 (m, 1H), 7.73 (t, *J* = 7.7 Hz, 2H), 7.58 (d, *J* = 9.9 Hz, 1H), 7.48 (t, *J* = 4.3 Hz, 4H), 7.28 (d, *J* = 9.9 Hz, 1H); ^13^C NMR (100 MHz, DMSO-*d_6_*) *δ* 158.22, 153.15, 145.78, 143.46, 142.56, 135.54, 135.00, 134.51, 130.00, 128.63, 126.17, 125.42. HRMS (EI) *m/z* for C_16_H_13_N_3_O_6_S_2_ [M + Na]^+^ calcd. 430.013798, found 430.014189.

##### 6-Oxo-1–(4-sulfamoylphenyl)-1,6-dihydropyridazin-3-yl 4-methylbenzenesulfonate (7d)

White powder (96%); ^1^H NMR (400 MHz, DMSO-*d_6_*) *δ* 7.91–7.87 (m, 4H), 7.59 − 7.42 (m, 7H), 7.27 (d, *J* = 9.9 Hz, 1H), 2.45 (s, 3H); ^13^C NMR (100 MHz, DMSO-*d_6_*) *δ* 158.21, 146.59, 145.76, 143.61, 142.52, 134.92, 131.46, 130.40, 130.35, 129.51, 128.72, 128.70, 126.13, 125.43, 124.64, 21.20. HRMS *m/z* for C_17_H_15_N_3_O_6_S_2_ [M + Na]^+^ calcd. 444.029448, found 444.029379.

##### 6-Oxo-1–(4-sulfamoylphenyl)-1,6-dihydropyridazin-3-yl 4-methoxybenzenesulfonate (7e)

White powder (91%); ^1^H NMR (400 MHz, DMSO-*d_6_*) *δ* 7.92 (d, *J* = 9.1 Hz, 2H), 7.89 − 7.81 (m, 4H), 7.60 − 7.43 (m, 5H), 7.19 (d, *J* = 9.1 Hz, 1H), 3.86 (s, 3H); ^13^C NMR (100 MHz, DMSO-*d_6_*) *δ* 167.22, 164.57, 158.24, 145.84, 143.51, 142.62, 132.21, 132.10, 131.23, 126.17, 125.52, 115.12, 56.09. HRMS *m/z* for C_17_H_15_N_3_O_7_S_2_ [M + Na]^+^ calcd 460.024362; found 460.024876.

##### 6-Oxo-1–(4-sulfamoylphenyl)-1,6-dihydropyridazin-3-yl 4-fluorobenzenesulfonate (7f)

White powder (89%); ^1^H NMR (400 MHz, DMSO-*d_6_*) *δ* 8.13 (dd, *J* = 8.9, 5.0 Hz, 1H), 7.94 (d, *J* = 8.9 Hz, 1H), 7.89 (dd, *J* = 8.6, 3.8 Hz, 2H), 7.62 − 7.47 (m, 6H), 7.30 − 7.20 (m, 2H); ^13^C NMR (100 MHz, DMSO-*d_6_*) *δ* 164.57, 158.24, 145.84, 145.73, 143.51, 142.62, 132.21, 132.10, 131.23, 126.17, 125.52, 115.12. HRMS (EI) *m/z* for C_16_H_12_FN_3_O_6_S_2_ [M + Na]^+^ calcd 448.004376, found 448.004591.

### Biological evaluations

#### COX-1, COX-2 and LOX inhibition assay

The ability of the examined pyridazine-based sulphonamide derivatives **3**, **5a-c** and **7a-f** to inhibit both COX‐1/COX‐2 has been *in vitro* investigated by the use of COX Inhibitor screening ELISA assay kit (cat. no. 560131, Cayman, USA) according to the supplier’s recommendations and as described previously.^35^ Besides, LOX inhibitory screening assay kit (Cat. No. 760700, Cayman, USA) was exploited to evaluate the 5-LOX inhibitory activity of the examined pyridazine-based sulphonamide derivatives **3**, **5a-c** and **7a-f** according to the manufacturer’s instructions and as previously reported.^48^

#### Determination of the CA inhibitory activities

The experimental methodology utilised to evaluate the CA inhibitory action of the pyridazine-based sulphonamide derivatives **3**, **5a-c** and **7a-f** disclosed here was previously described.^49–53^

#### *In vivo* assays

The experimental procedures adopted to perform the acetic acid-induced writhing assay for the analgesic activities,^45^ Paw edoema assay for the anti-inflammatory activities,^46^ as well as the acute ulcerogenic test^47^ have been carried out as described previously.

#### Biochemical determination for certain inflammatory mediators

ELISA assays were used to assess the serum level for the inflammatory mediators TNF-α (Kit Cat. No MBS355371, MyBioSource, USA), and IL-1β (Kit Cat. No MBS8825017, MyBioSource, USA) based on the manufacture instructions.^54^

### Molecular Modelling

For the computational docking study, crystal structures of CA I, II, IX and XII (PDBs: 6Y00, 3K34, 5FL4 and 5LL5, respectively^55–58^) have been downloaded from Protein Data Bank^59^ and prepared using the Protein Preparation Wizard tool implemented in the Schrödinger suite. The detailed procedures exploited in this analysis are previously reported.^54^

## Supplementary Material

Supplemental Material
