# Supplementary material for: Identification of new 4-(6-oxopyridazin-1-yl)benzenesulfonamides as multi-target anti-inflammatory agents targeting carbonic anhydrase, COX-2 and 5-LOX enzymes: synthesis, biological evaluations and modelling insights
Source: J Enzyme Inhib Med Chem. 2023 Apr 20;38(1):2201407. doi: 10.1080/14756366.2023.2201407 (PMC10120535; doi:10.1080/14756366.2023.2201407)
Supplement: Supplemental Material [file IENZ_A_2201407_SM7928.pdf]

Supporting information

Identification of new 4-(6-oxopyridazin-1-yl)benzenesulfonamides as multi-target anti-inflammatory agents targeting carbonic anhydrase, COX-2 and 5-LOX enzymes: Synthesis, biological evaluations and modeling insights

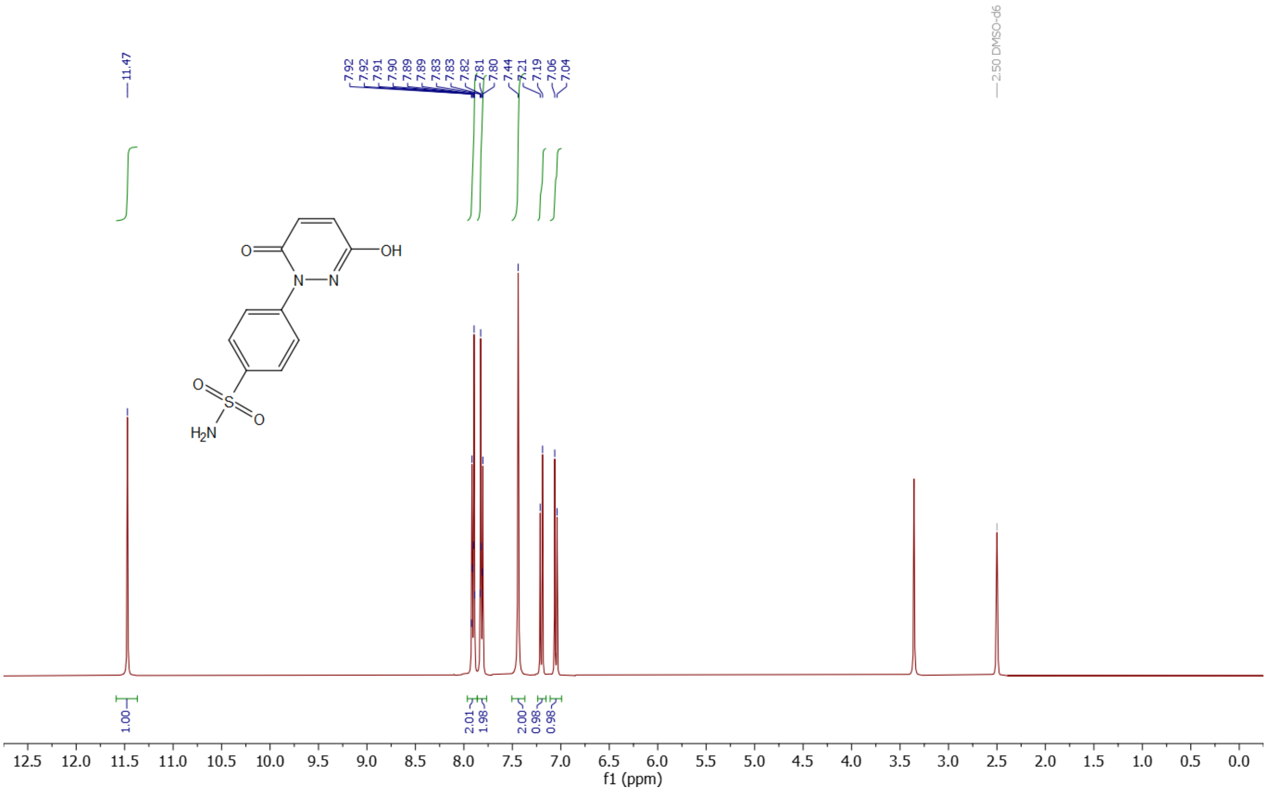

Figure **1**. ^{1}H NMR (400 MHz, DMSO-d_6) spectrum of compound **3**.

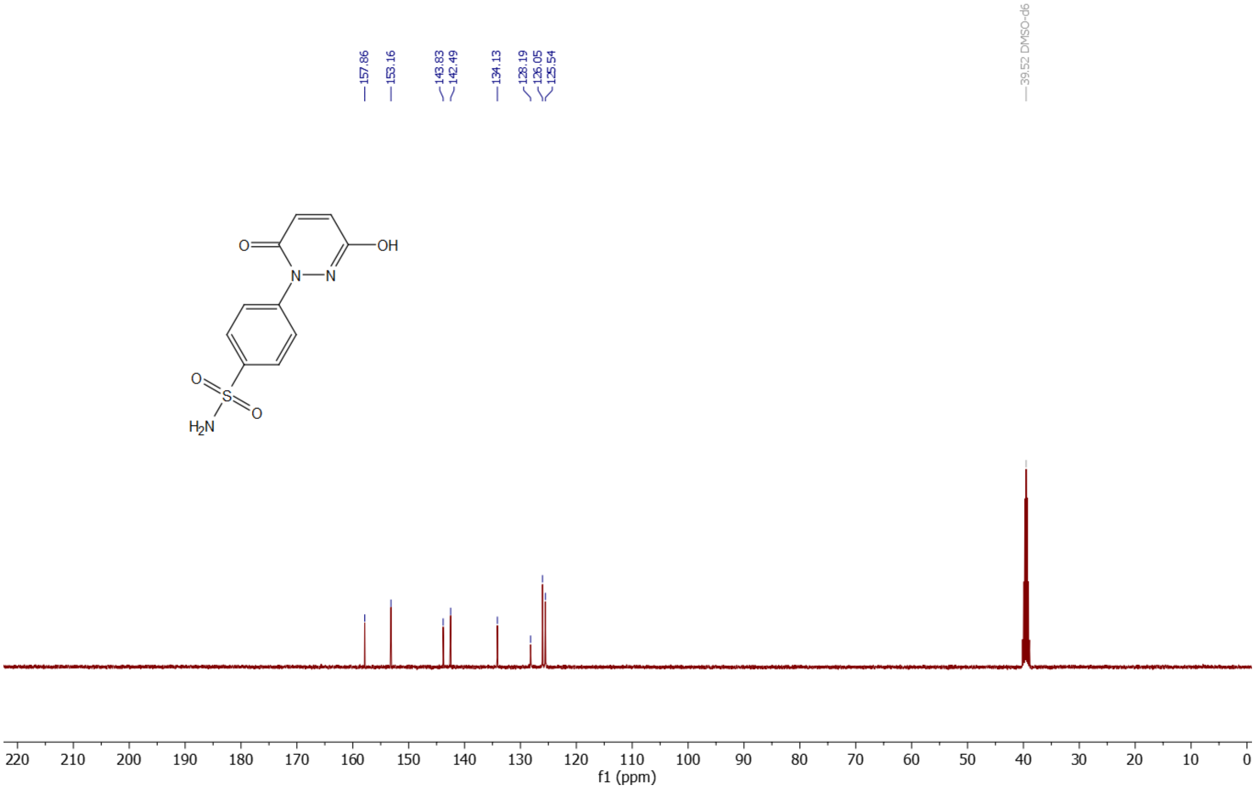

Figure **2**. ^{13}C (400 MHz, DMSO-d_6) spectrum of compound **3**.

Mass Spectrum List Report

**Analysis Info**		Acquisition Date	12/29/2021 12:29:02 PM
Analysis Name	Z:\FTICR-MS\MS-1\Data\2021\apexdata122921\ME-5003_pos_000001.d		
Method		Operator	COSMIC
Sample Name	ME-5003	Instrument	apex-Qe
Comment	ME-5003 C10H9N3O4S Na+		

Sample Name	ME-5003			
Exact Mass of	C10H9N3O4S Na+	=	290.020598	m/z
Mass observed		=	290.020620	m/z

Difference < 1.0 ppm

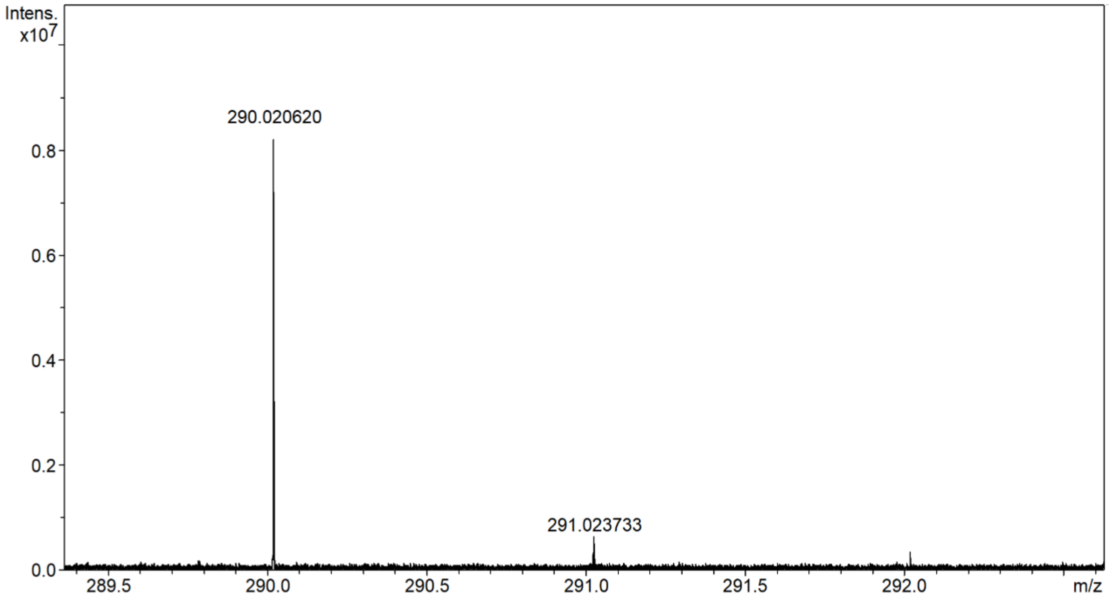

HRMS spectrum of compound **3**.

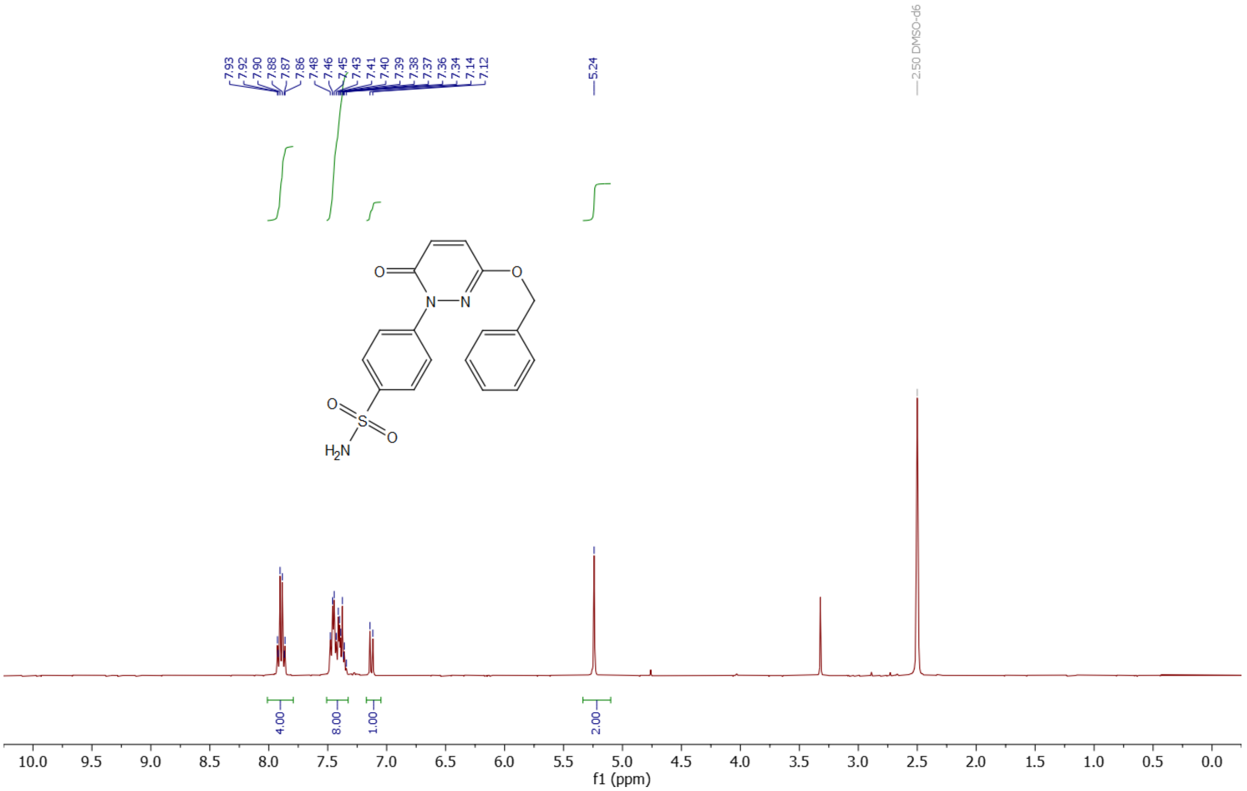

Figure **3**. ^{1}H NMR (400 MHz, DMSO-d_6) spectrum of compound **5a**.

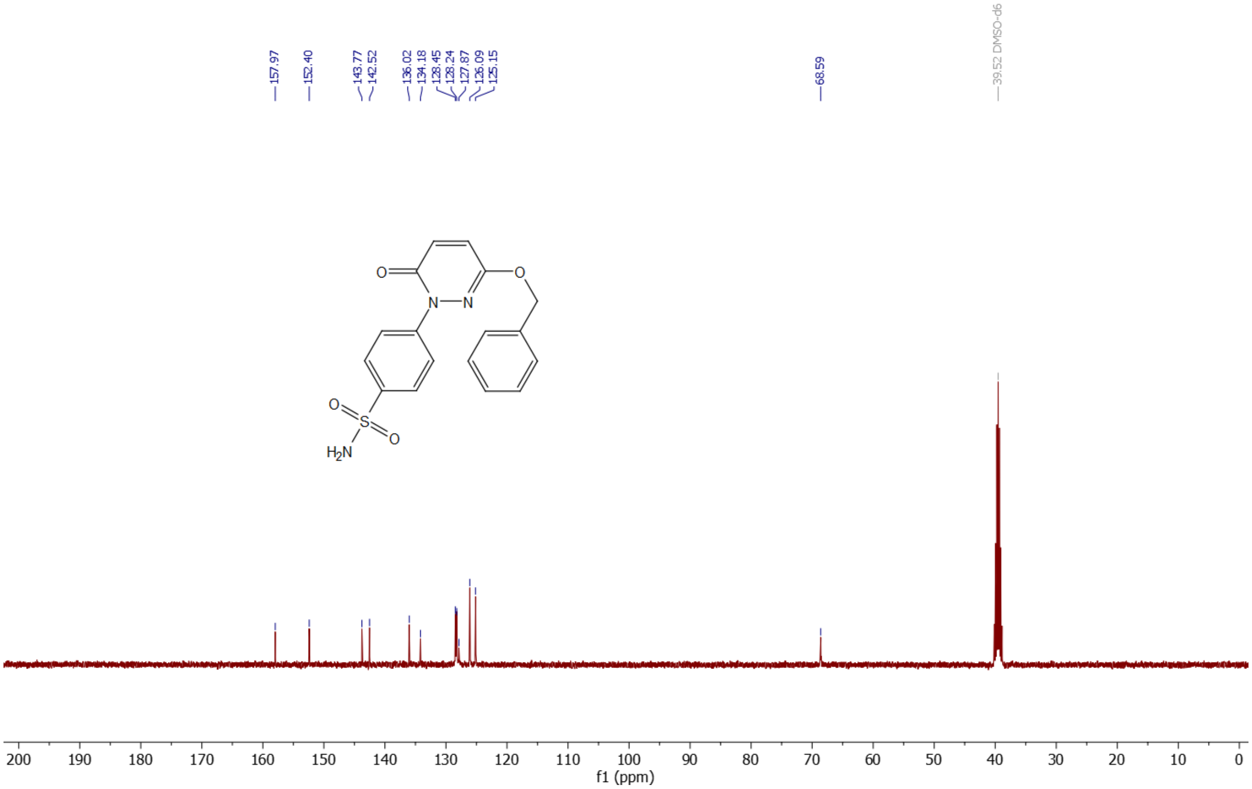

Figure **4**. ^{13}C (400 MHz, DMSO-*d_6*) spectrum of compound **5a**.

Mass Spectrum List Report

**Analysis Info**		Acquisition Date	12/29/2021 12:17:50 PM
Analysis Name	Z:\FTICR-MS\MS-1\Data\2021\apexdata122921\ME-5001_pos_000001.d		
Method		Operator	COSMIC
Sample Name	ME-5001	Instrument	apex-Qe
Comment	ME-5001 C17H15N3O4S Na+		

Sample Name ME-5001

Exact Mass of C17H15N3O4S Na+ = 380.057548 m/z

Mass observed = 380.067428 m/z

Difference < 1.0 ppm

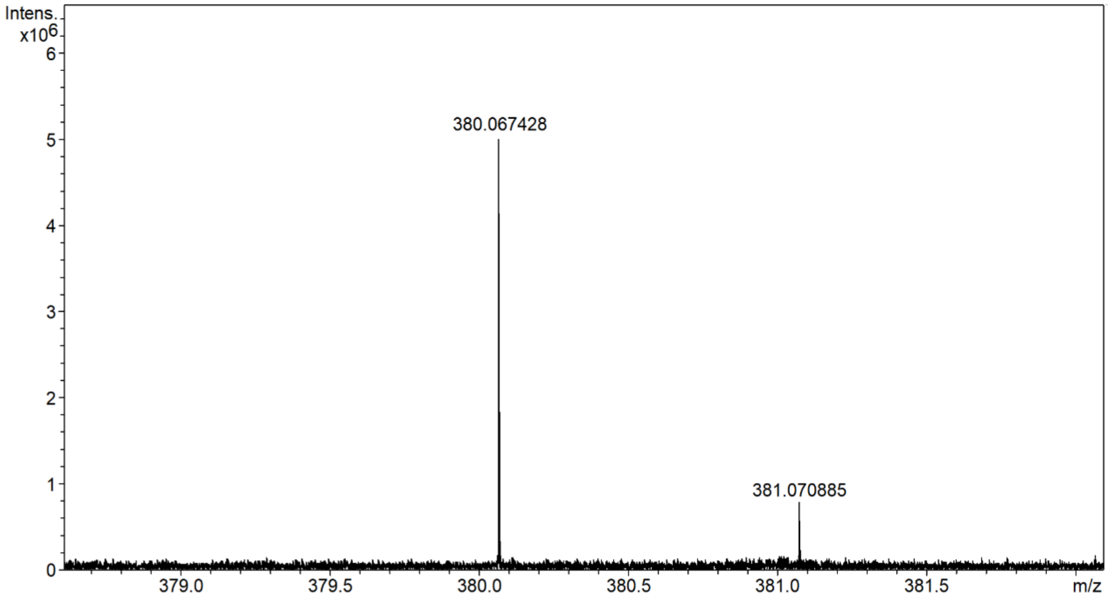

HRMS spectrum of compound **5a**.

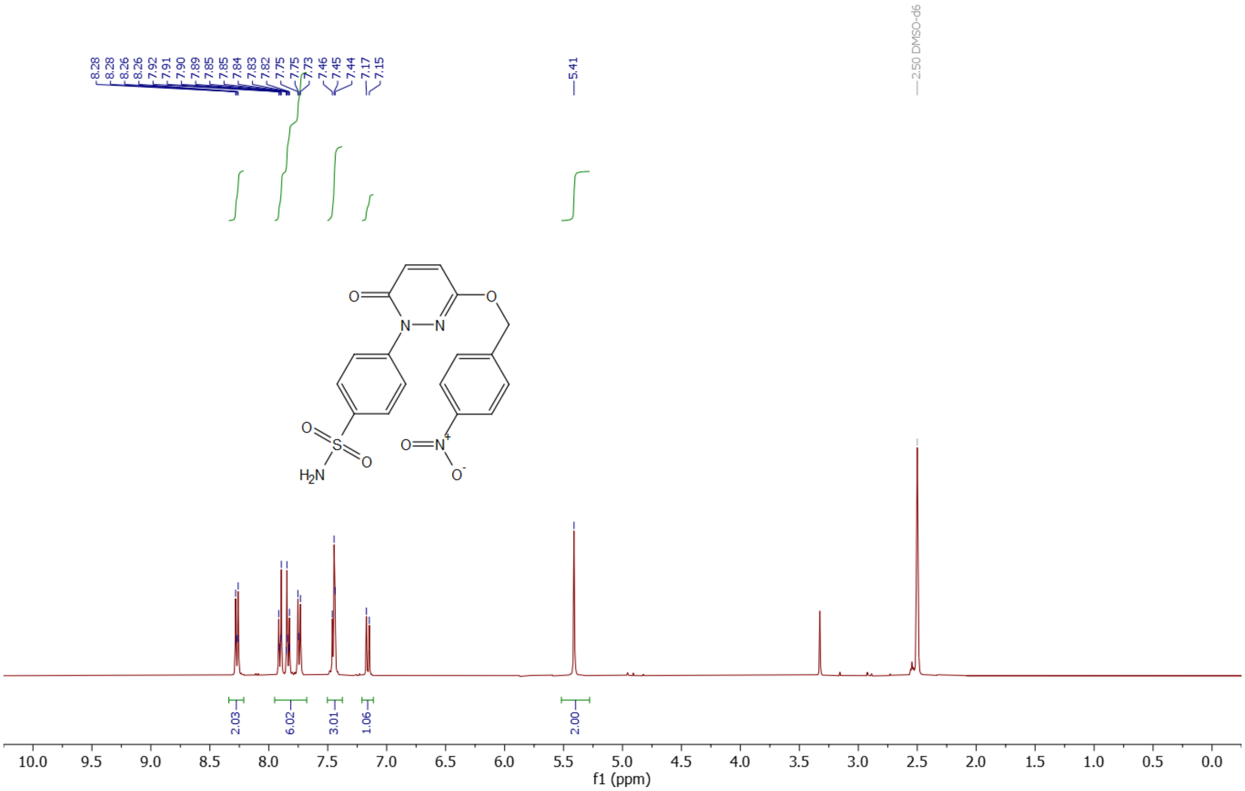

Figure **5**. ^{1}H NMR (400 MHz, DMSO-d_6) spectrum of compound **5b**.

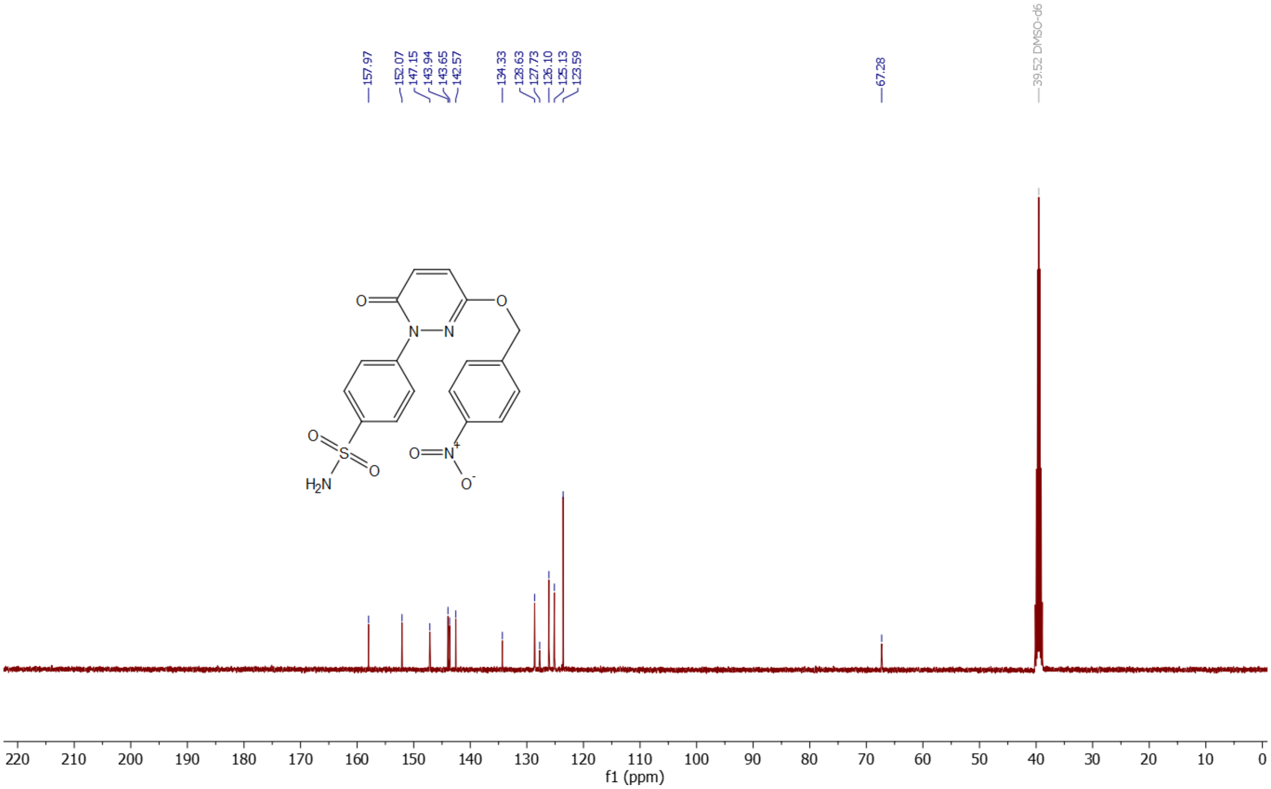

Figure **6**. ^{13}C (400 MHz, DMSO-d_6) spectrum of compound **5b**.

Mass Spectrum List Report

Analysis Info

Analysis Name	Z:\FTICR-MS\MS-1\Data\2021\apexdata122921\ME-5005_pos_000001.d	Acquisition Date	12/29/2021 12:37:55 PM
Method		Operator	COSMIC
Sample Name	ME-5005	Instrument	apex-Qe
Comment	ME-5005 C17H14N4O6S Na+		

Sample Name	ME-5005			
Exact Mass of	C17H14N4O6S Na+	=	425.052626	m/z
Mass observed		=	425.052865	m/z

Difference < 1.0 ppm

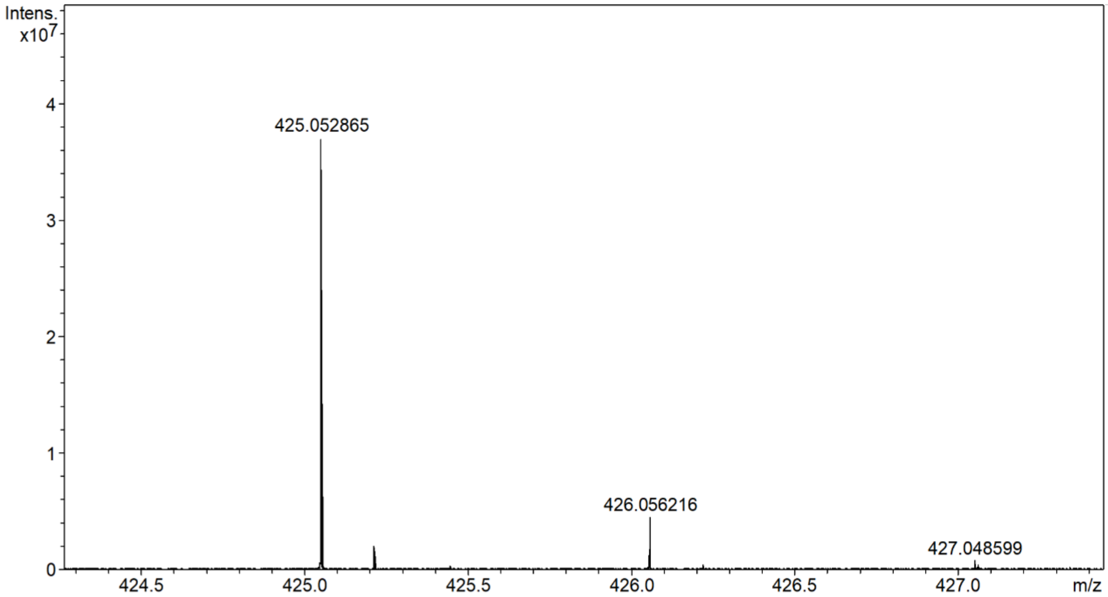

HRMS spectrum of compound **5b**.

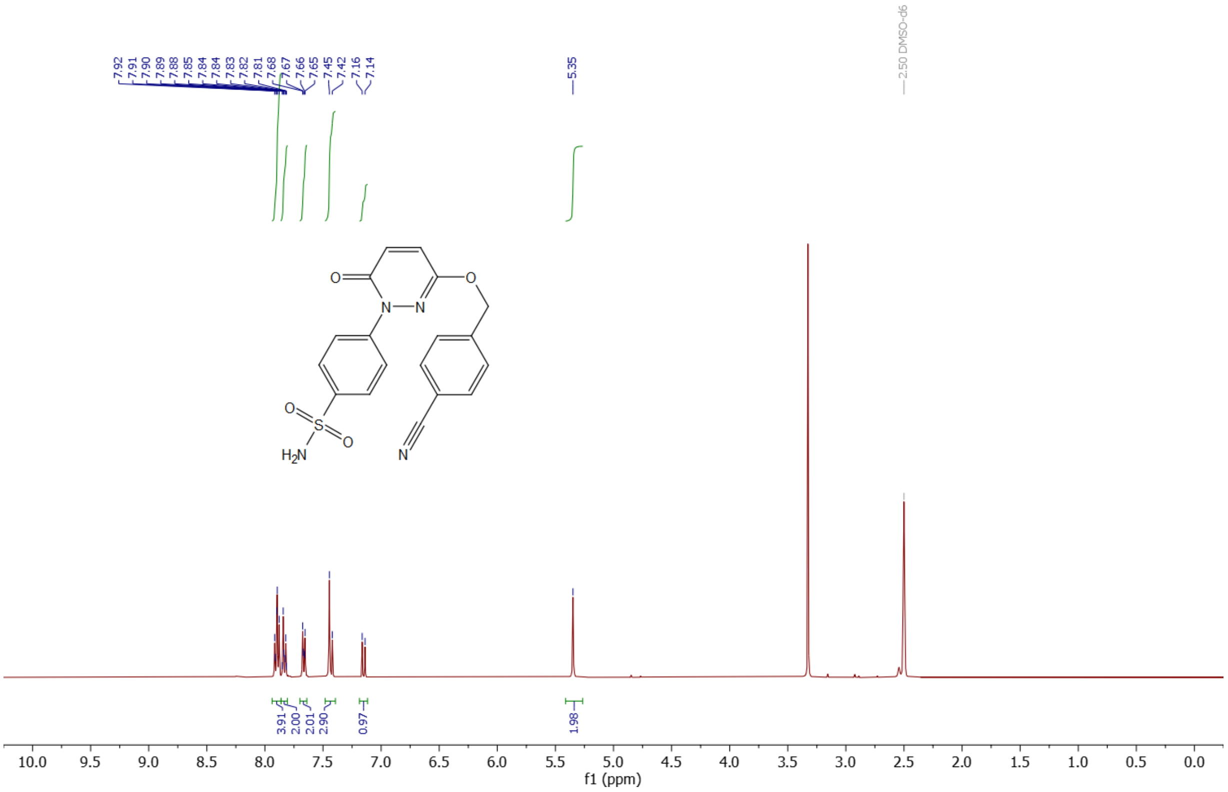

Figure **7**. ^{1}H NMR (400 MHz, DMSO-d_6) spectrum of compound **5c**.

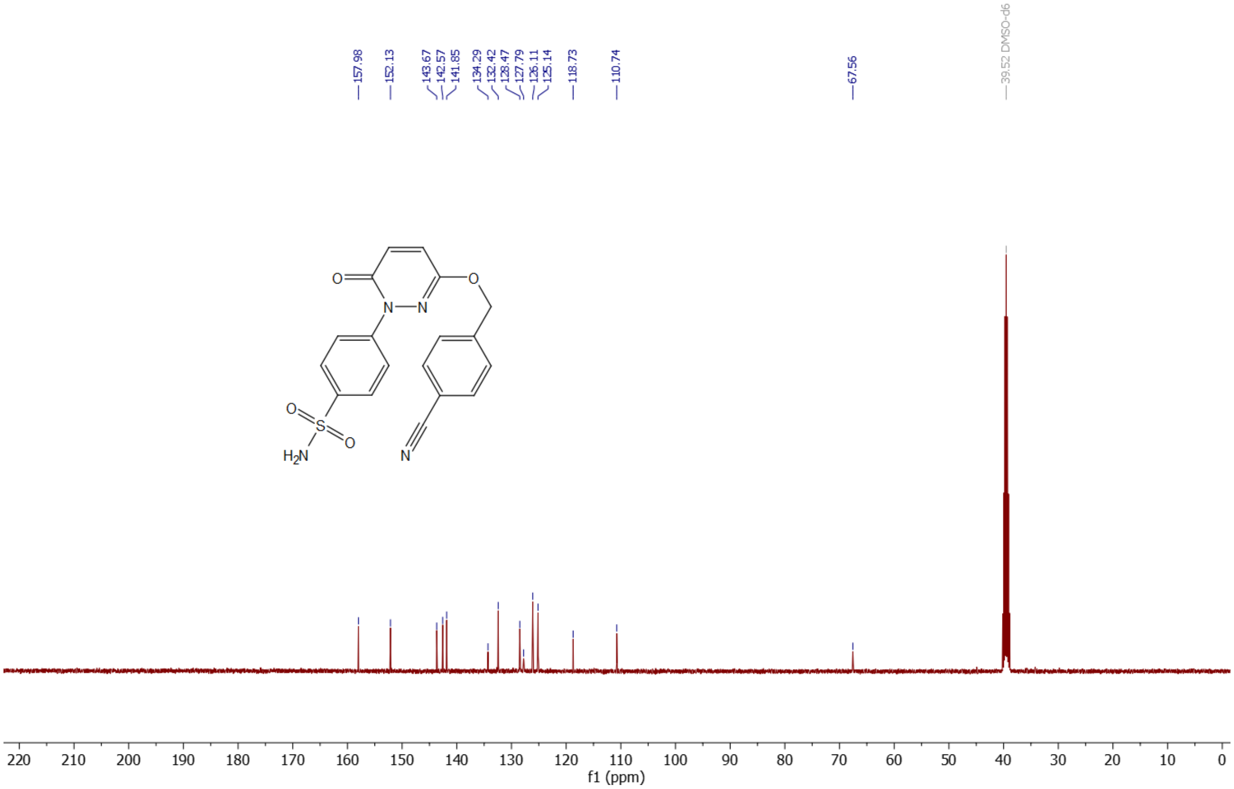

Figure **8**. ^{13}C (400 MHz, DMSO-d_6) spectrum of compound **5c**.

Mass Spectrum List Report

Analysis Info

Analysis Name	Z:\FTICR-MS\MS-1\Data\2021\apexdata122921\ME-5004_pos_000001.d	Acquisition Date	12/29/2021 12:33:12 PM
Method		Operator	COSMIC
Sample Name	ME-5004	Instrument	apex-Qe
Comment	ME-5004 C18H14N4O4S Na+		

Sample Name ME-5004

Exact Mass of C18H14N4O4S Na+ = 405.062797 m/z

Mass observed = 405.062819 m/z

Difference < 1.0 ppm

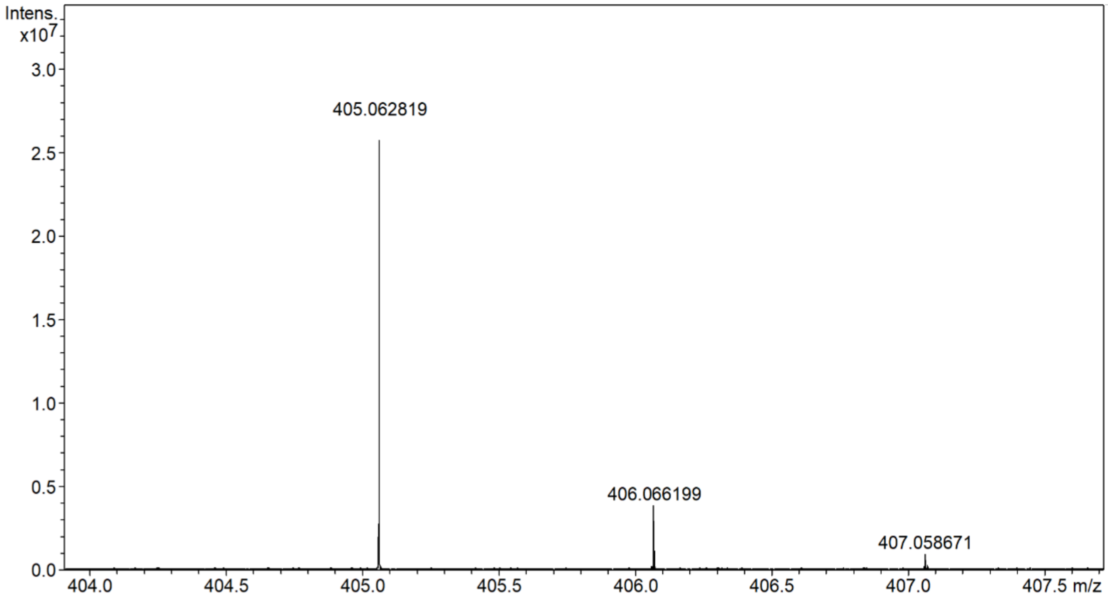

HRMS spectrum of compound **5c**.

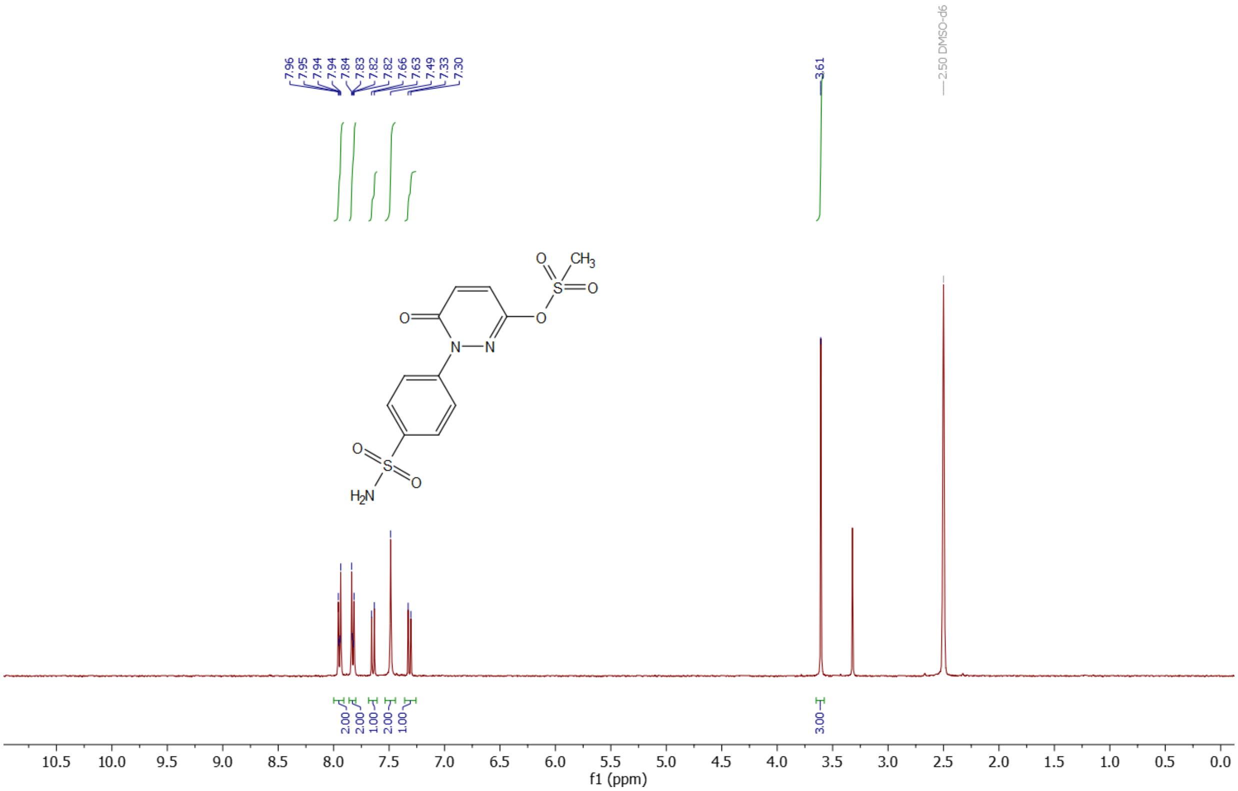

Figure **9**. ^{1}H NMR (400 MHz, DMSO-d_6) spectrum of compound **7a**.

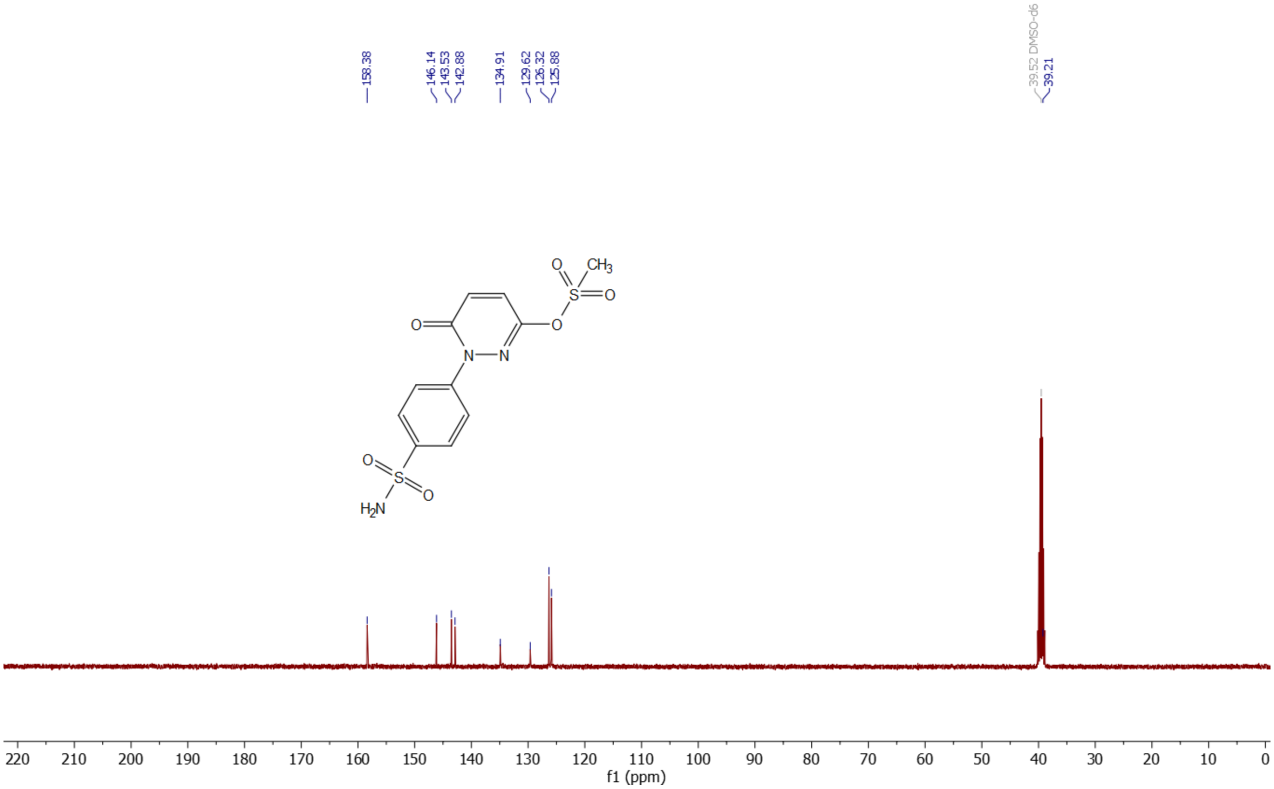

Figure **10**. ^{13}C (400 MHz, DMSO-d_6) spectrum of compound **7a**.

Mass Spectrum List Report

Analysis Info

		Acquisition Date	12/29/2021 12:43:02 PM
Analysis Name	Z:\FTICR-MS\MS-1\Data\2021\apexdata122921\ME-5006_pos_000001.d		
Method		Operator	COSMIC
Sample Name	ME-5006	Instrument	apex-Qe
Comment	ME-5006 C11H11N3O6S2 Na+		

Sample Name	ME-5006			
Exact Mass of	C11H11N3O6S2 Na+	=	367.998148	m/z
Mass observed		=	367.998341	m/z

Difference < 1.0 ppm

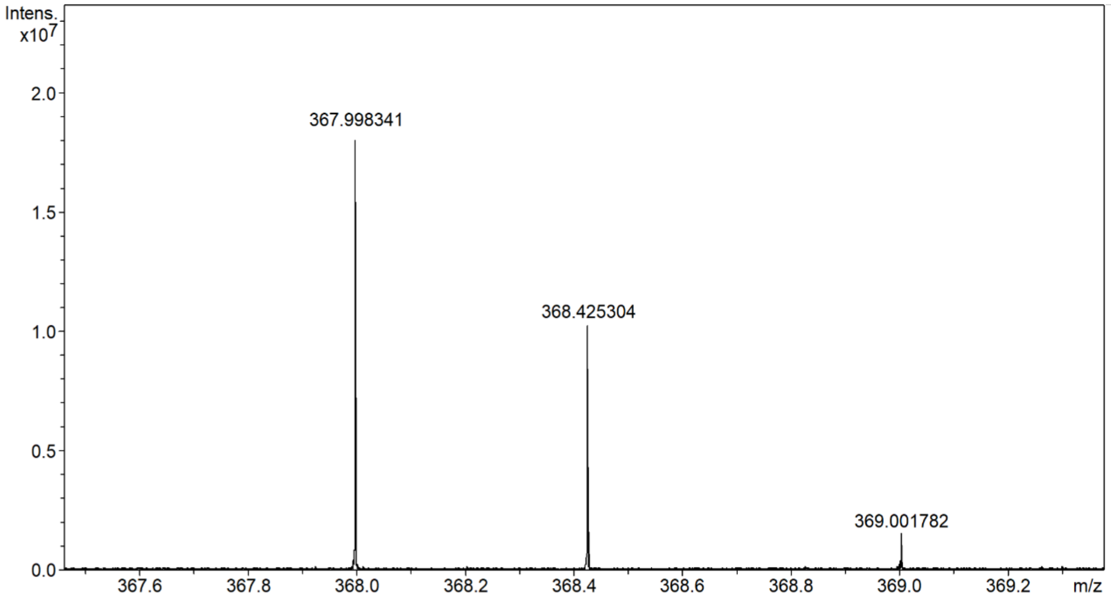

HRMS spectrum of compound **7a**.

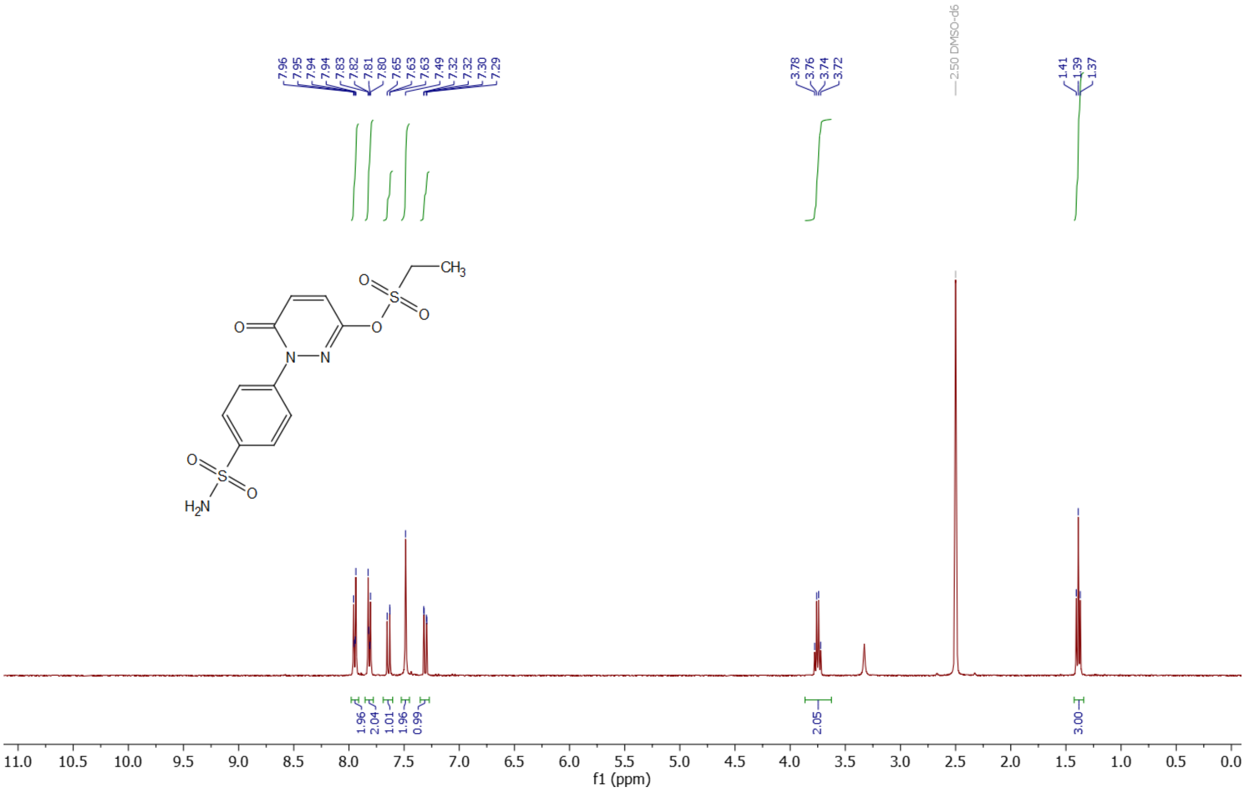

Figure **11**. ^{1}H NMR (400 MHz, DMSO-d_6) spectrum of compound **7b**.

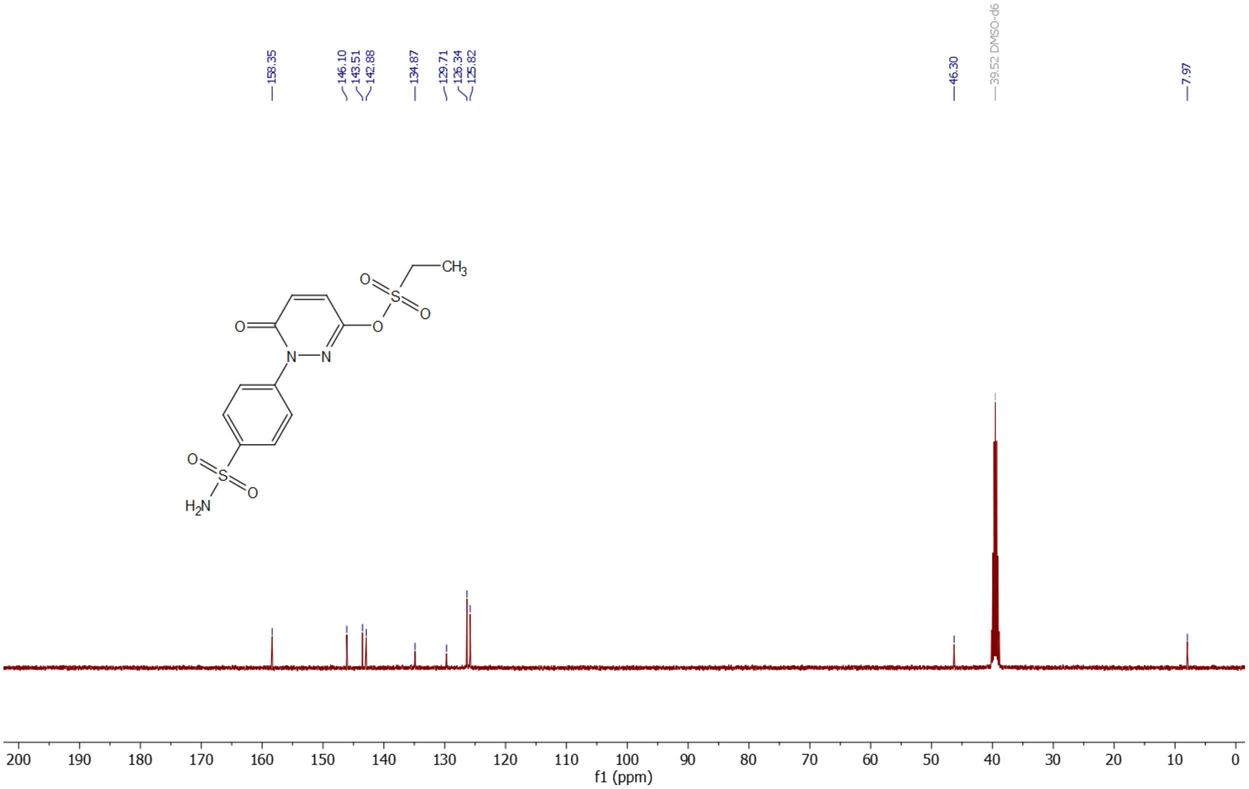

Figure **12**. ^{13}C (400 MHz, DMSO-d_6) spectrum of compound **7b**.

Mass Spectrum List Report

Analysis Info

Analysis Name	Z:\FTICR-MS\MS-1\Data\2021\apexdata122921\ME-5008_pos_000001.d	Acquisition Date	12/29/2021 12:52:25 PM
Method		Operator	COSMIC
Sample Name	ME-5008	Instrument	apex-Qe
Comment	ME-5008 C12H13N3O6S2 Na+		

Sample Name	ME-5008			
Exact Mass of	C12H13N3O6S2 Na+	=	382.013798	m/z
Mass observed		=	382.013848	m/z

Difference < 1.0 ppm

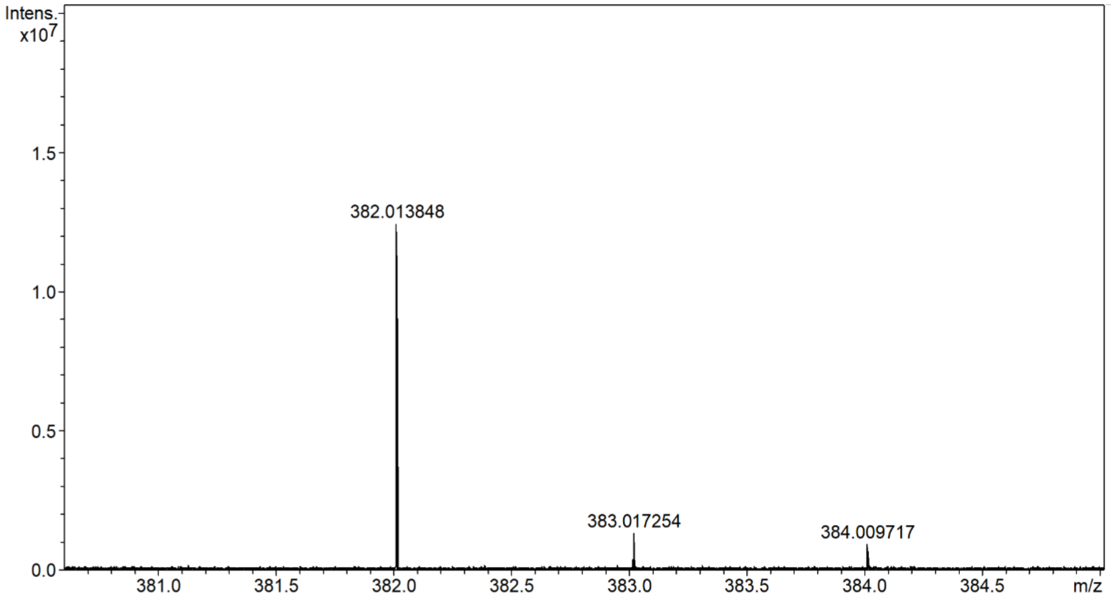

HRMS spectrum of compound **7b**.

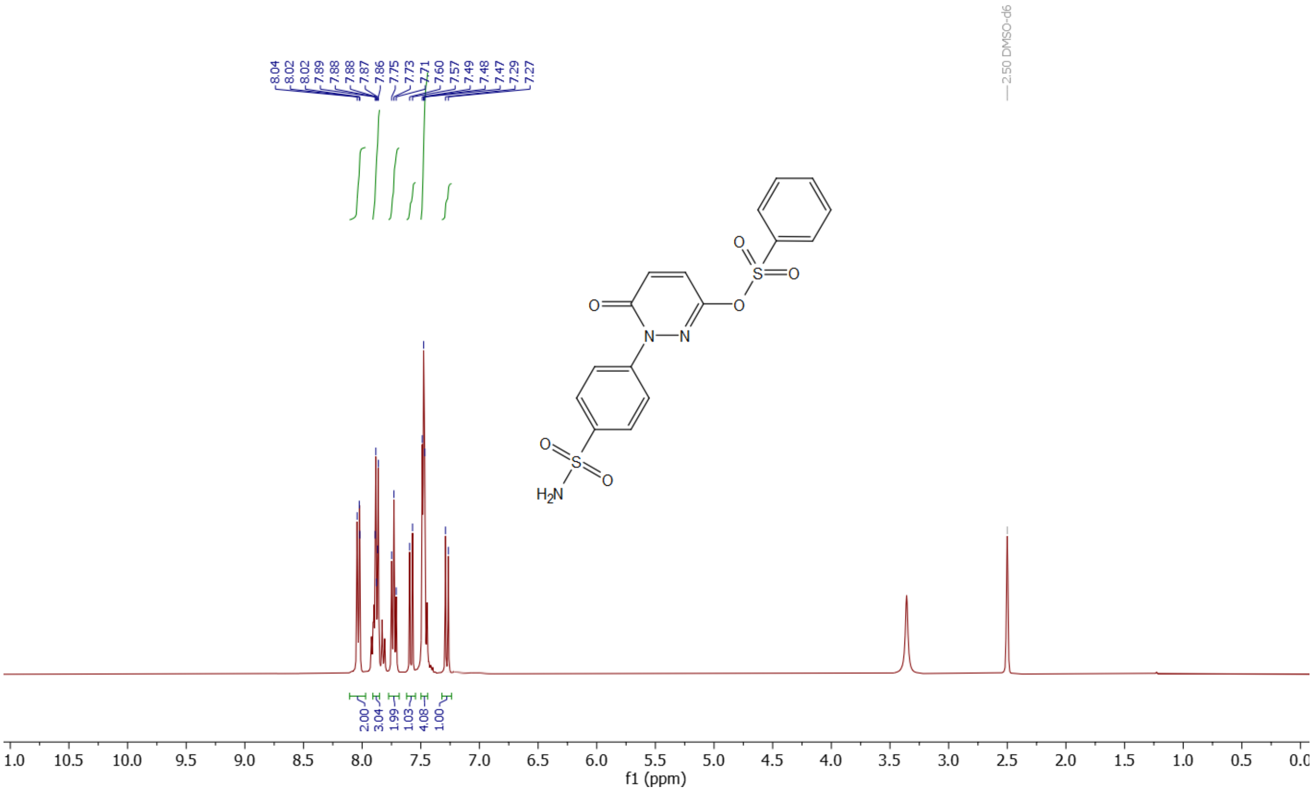

Figure **13**. ^{1}H NMR (400 MHz, DMSO-d_6) spectrum of compound **7c**.

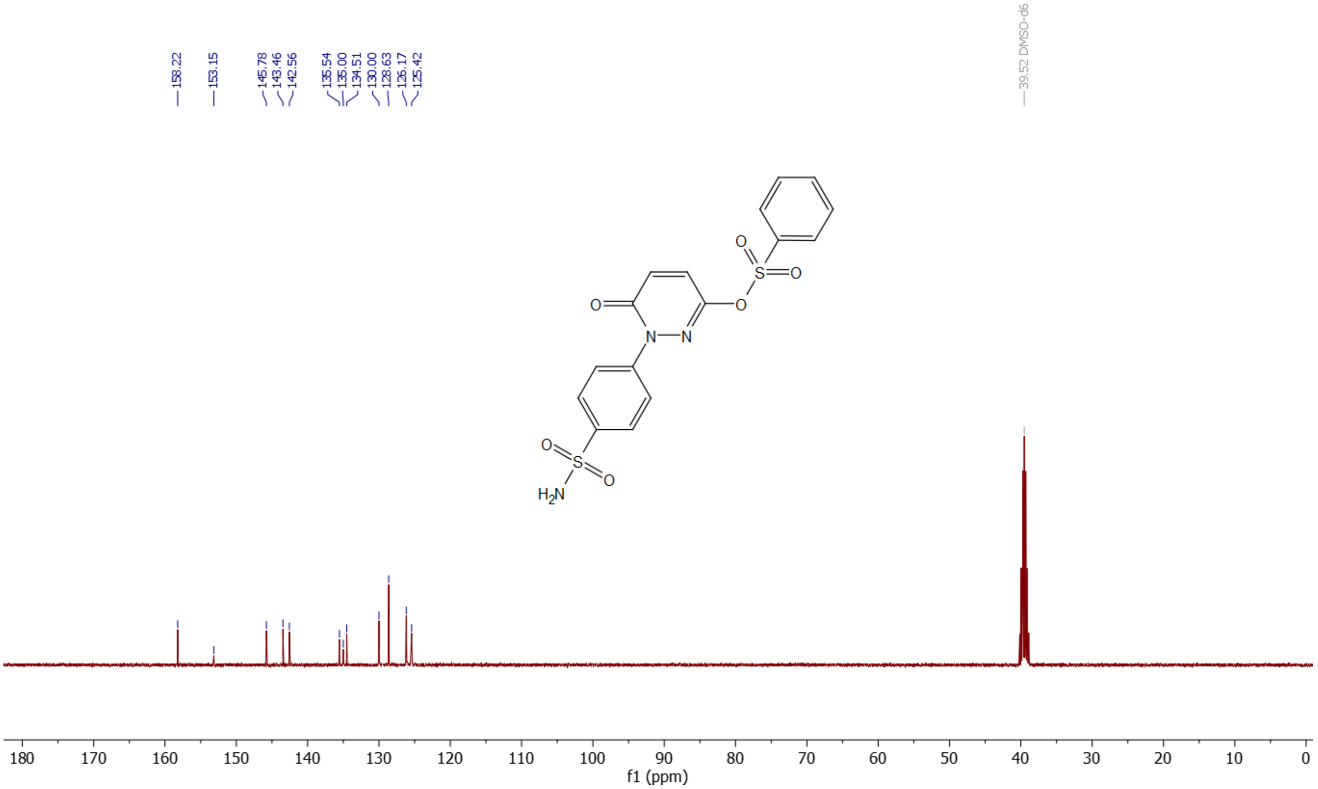

Figure **14**. ^{13}C (400 MHz, DMSO-d_6) spectrum of compound **7c**.

Mass Spectrum List Report

Analysis Info

		Acquisition Date	12/29/2021 1:02:03 PM
Analysis Name	Z:\FTICR-MS\MS-1\Data\2021\apexdata122921\ME-5010_pos_000001.d		
Method		Operator	COSMIC
Sample Name	ME-5010	Instrument	apex-Qe
Comment	ME-5010 C16H13N3O6S2 Na+		

Sample Name ME-5010

Exact Mass of C16H13N3O6S2 Na+ = 430.013798 m/z

Mass observed = 430.014189 m/z

Difference < 1.0 ppm

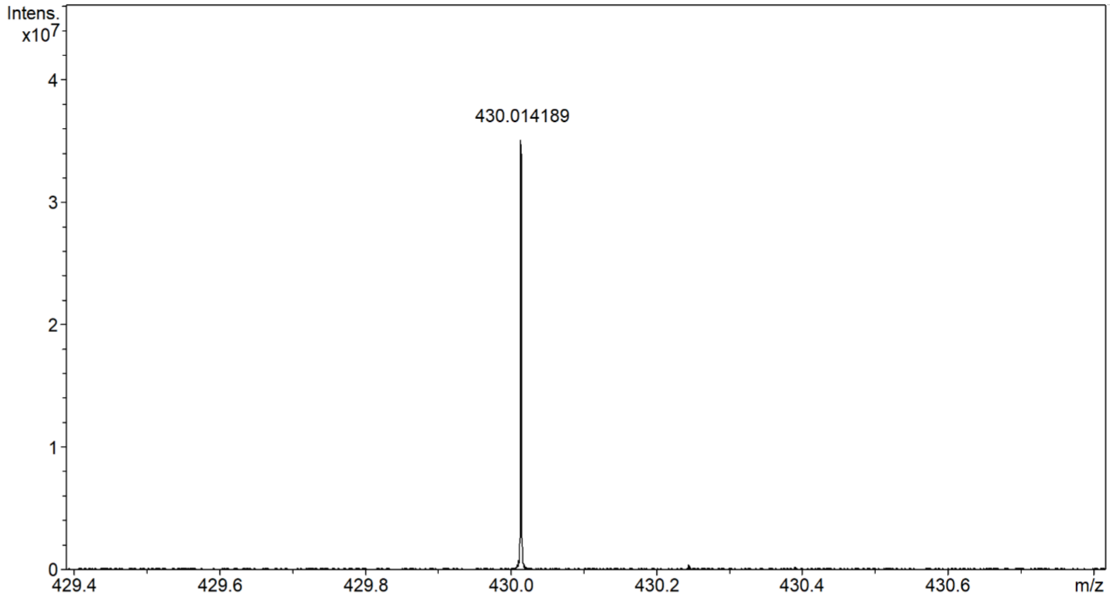

HRMS spectrum of compound **7c**.

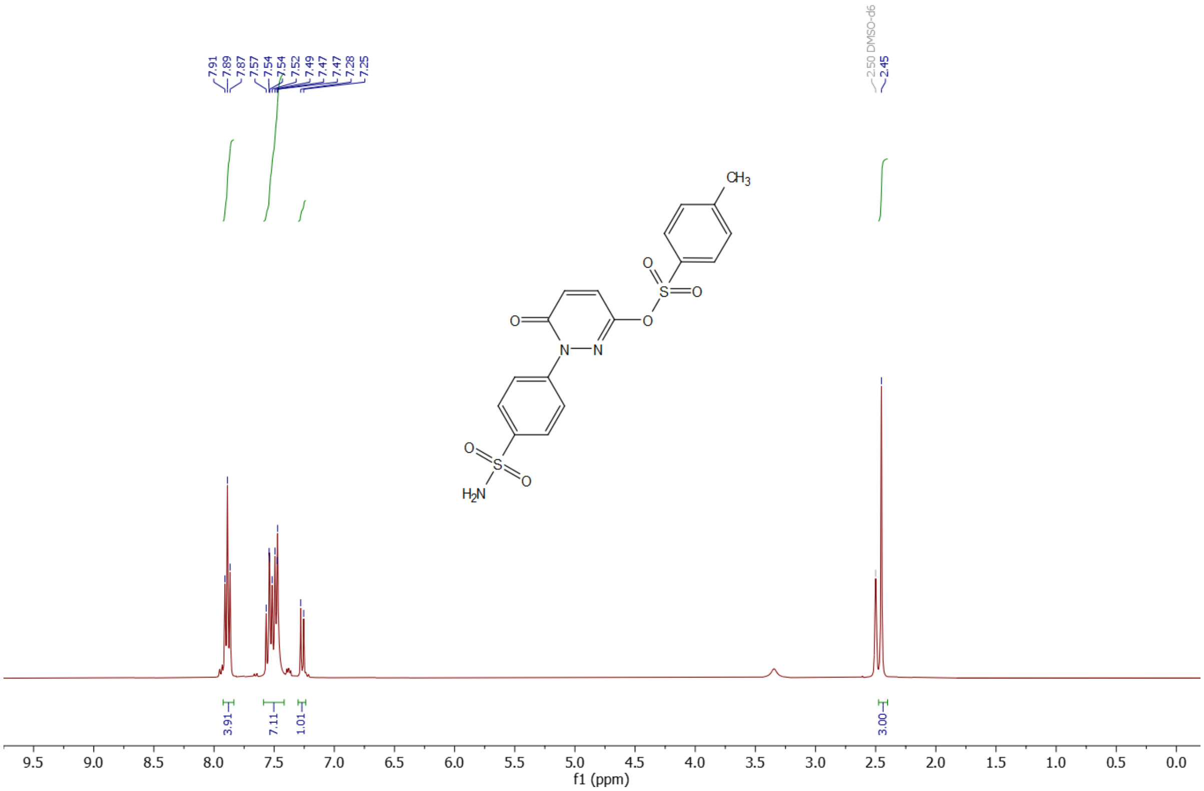

Figure **15**. ^{1}H NMR (400 MHz, DMSO-d_6) spectrum of compound **7d**.

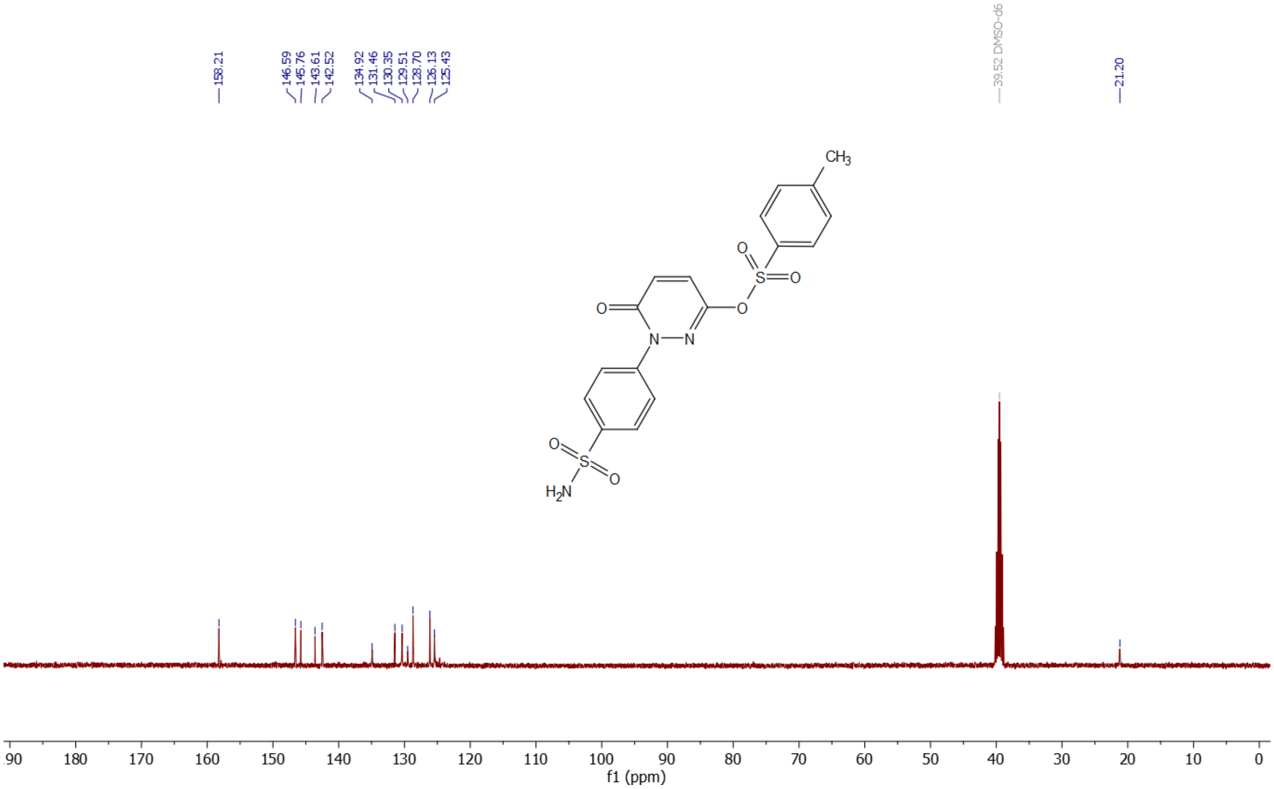

Figure **16**. ^{13}C (400 MHz, DMSO-d_6) spectrum of compound **7d**.

Mass Spectrum List Report

Analysis Info

Analysis Name	Z:\FTICR-MS\MS-1\Data\2021\apexdata122921\ME-5002_pos_000001.d	Acquisition Date	12/29/2021 12:23:12 PM
Method		Operator	COSMIC
Sample Name	ME-5002	Instrument	apex-Qe
Comment	ME-5002 C17H15N3O6S2 Na+		

Sample Name ME-5002

Exact Mass of C17H15N3O6S2 Na+ = 444.029448 m/z

Mass observed = 444.029379 m/z

Difference < 1.0 ppm

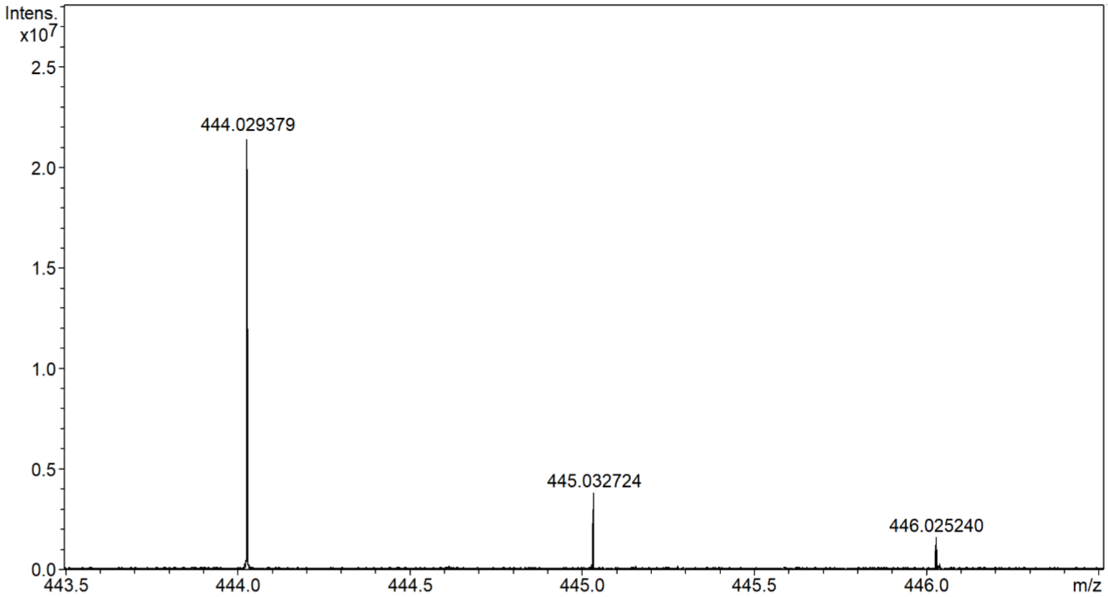

HRMS spectrum of compound **7d**.

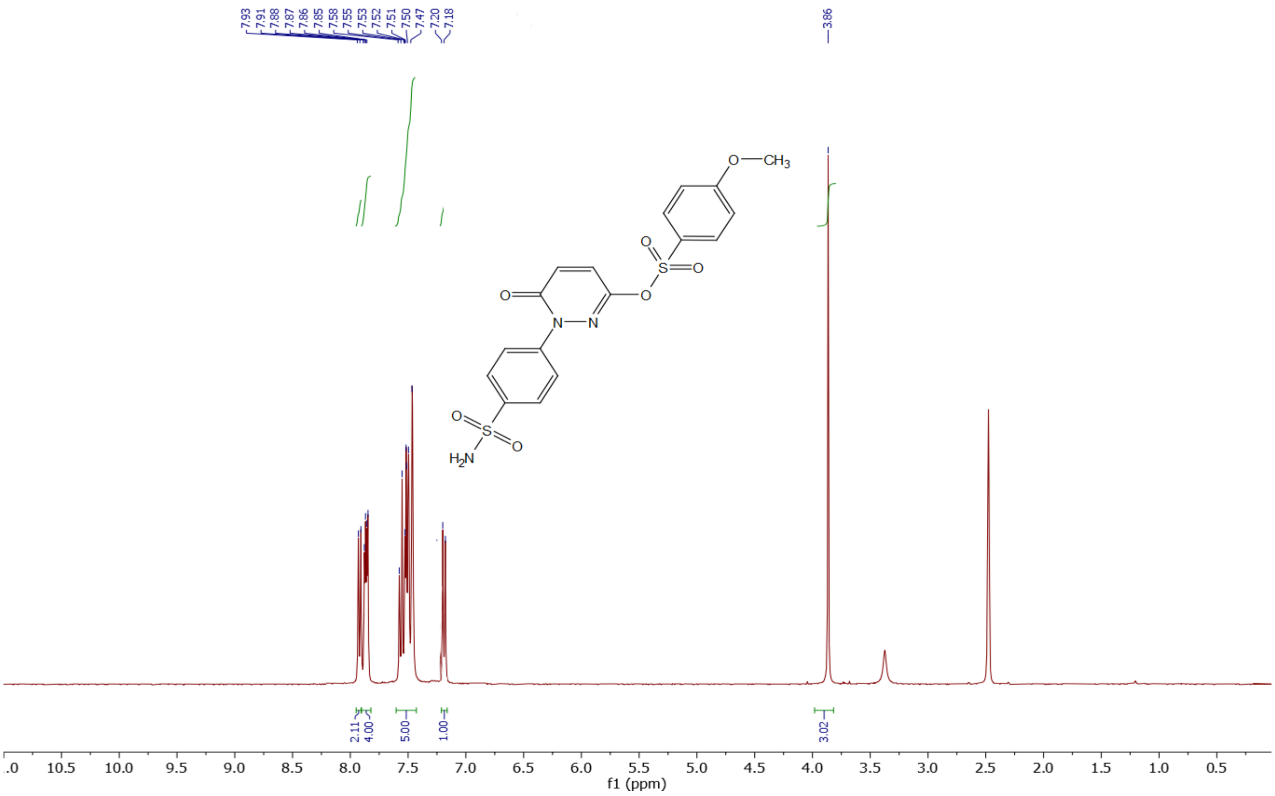

Figure **17**. ^{1}H NMR (400 MHz, DMSO-d_6) spectrum of compound **7e**.

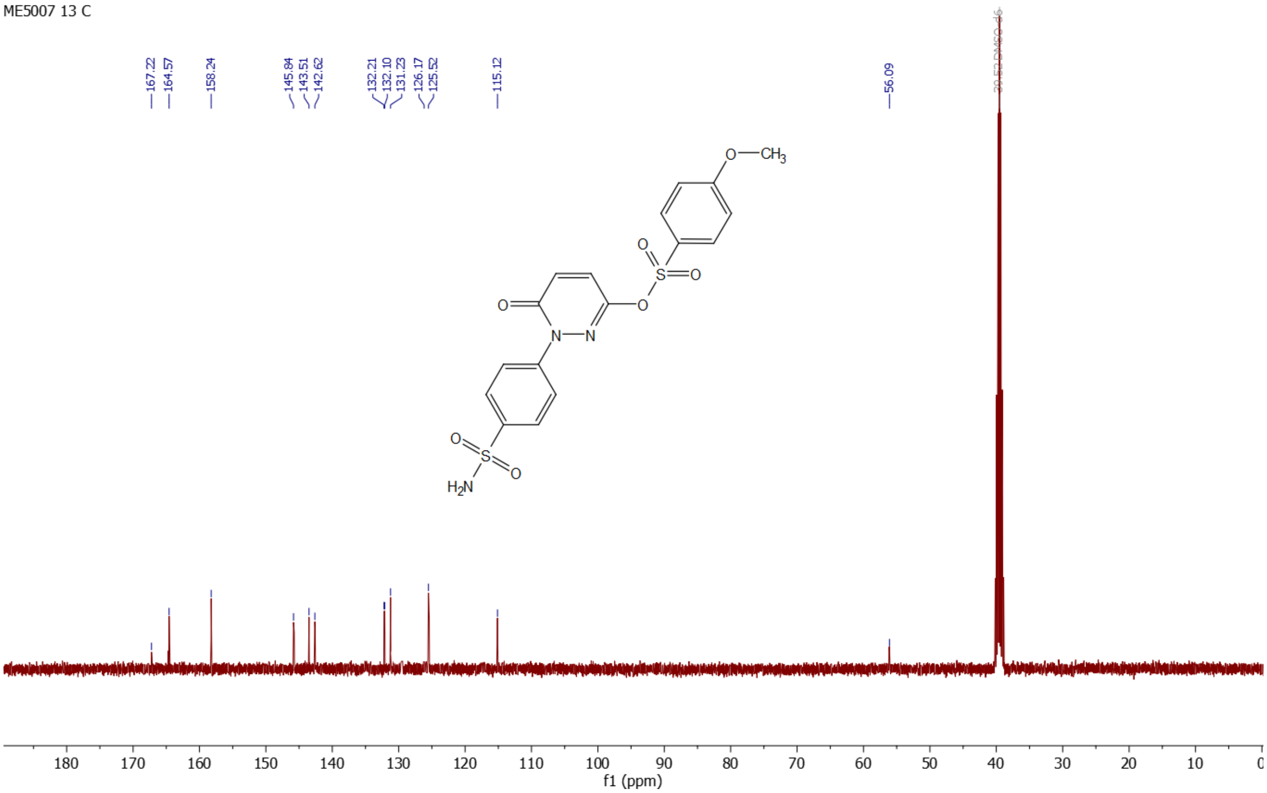

Figure **18**. ^{13}C (400 MHz, DMSO-d_6) spectrum of compound **7e**.

Mass Spectrum List Report

Analysis Info

Analysis Name	Z:\FTICR-MS\MS-1\Data\2021\apexdata122921\ME-5009_pos_000001.d	Acquisition Date	12/29/2021 12:57:04 PM
Method		Operator	COSMIC
Sample Name	ME-5009	Instrument	apex-Qe
Comment	ME-5009 C17H15N3O7S2 Na+		

Sample Name	ME-5009			
Exact Mass of	C17H15N3O7S2 Na+	=	460.024362	m/z
Mass observed		=	460.024876	m/z

Difference < 1.0 ppm

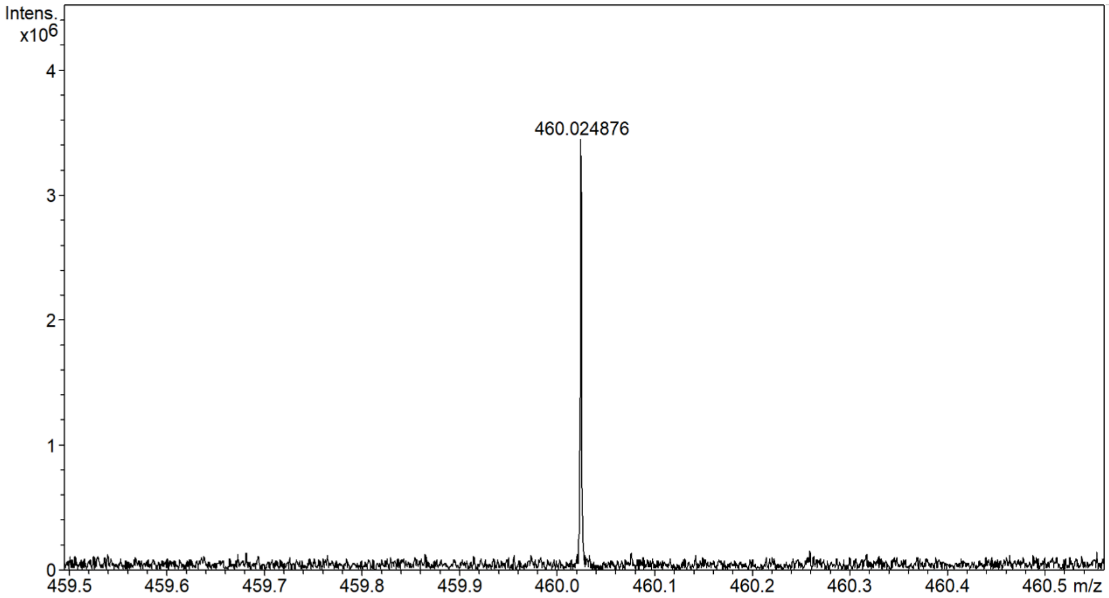

HRMS spectrum of compound **7e**.

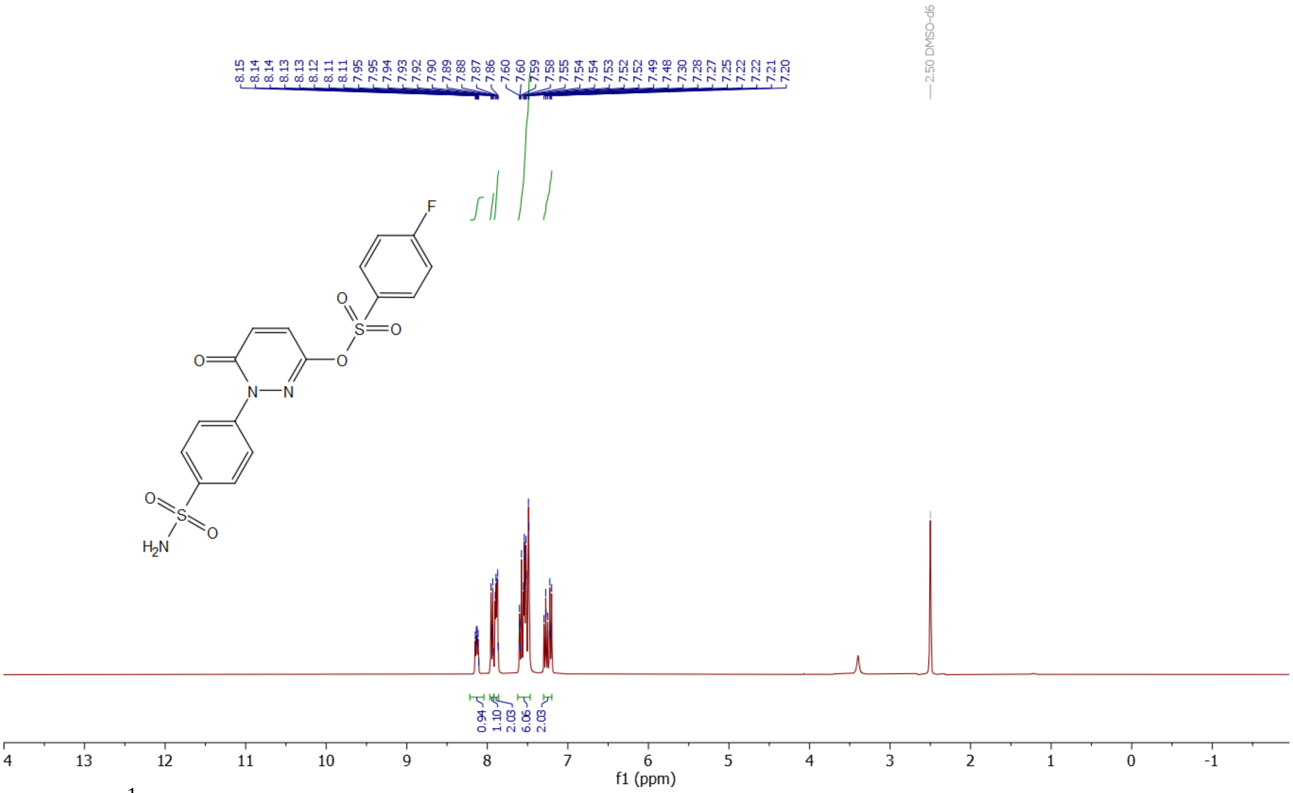

Figure **5**. ^{1}H NMR (400 MHz, DMSO-d_6) spectrum of compound **7f**.

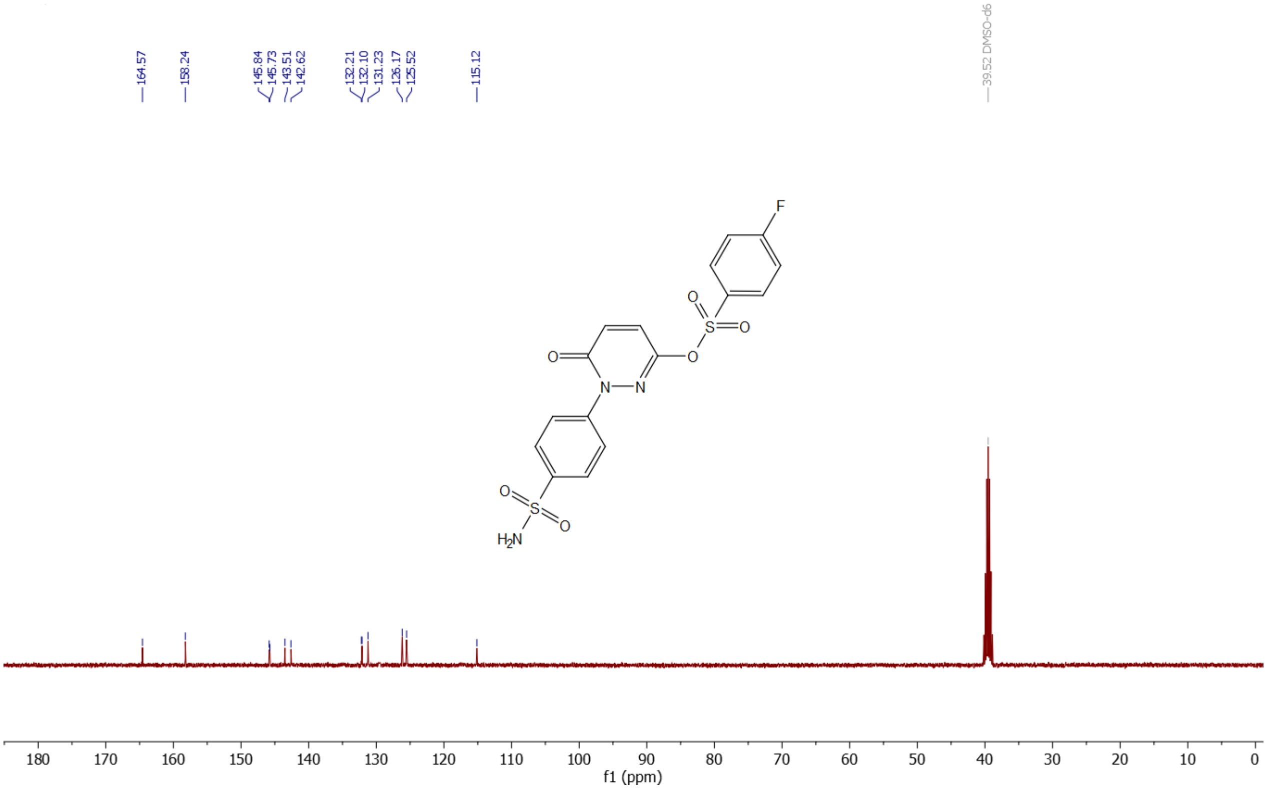

Figure **6**. ^{13}C (400 MHz, DMSO-d_6) spectrum of compound **7f**.

Mass Spectrum List Report

**Analysis Info**		Acquisition Date	12/29/2021 12:47:45 PM
Analysis Name	Z:\FTICR-MS\MS-1\Data\2021\apexdata122921\ME-5007_pos_000001.d		
Method		Operator	COSMIC
Sample Name	ME-5007	Instrument	apex-Qe
Comment	ME-5007 C16H12FN3O6S2 Na+		

Sample Name	ME-5007			
Exact Mass of	C16H12FN3O6S2 Na+	=	448.004376	m/z
Mass observed		=	448.004591	m/z

Difference < 1.0 ppm

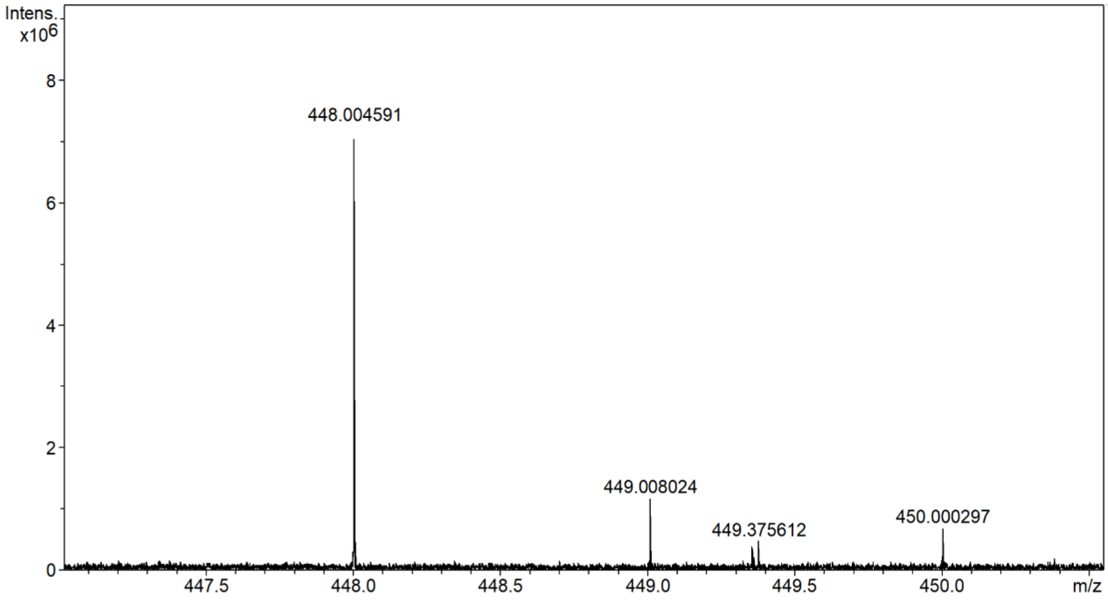

HRMS spectrum of compound **7f**.
